# Burden, trends and projections of liver cancer in China and G20 countries: a comparative study based on the global burden of disease database 2021

**DOI:** 10.3389/fpubh.2025.1668750

**Published:** 2026-01-16

**Authors:** Yi-fan He, Ya-meng Liu, Bing-nan Ren, Jing An, Yu-pei Wu, Jian-qun Zhao, Yin-ling Ma, Wei He, Hui-zhen Wu

**Affiliations:** 1 Department of Pharmacy, Hebei General Hospital, Shijiazhuang, China; 2 Hebei Key Laboratory of Clinical Pharmacy, Hebei General Hospital, Shijiazhuang, China; 3 Department of Cardiology, The Second Hospital of Hebei Medical University, Shijiazhuang, China; 4 Department of Hepatobiliary Surgery, The Second Hospital of Hebei Medical University, Shijiazhuang, China

**Keywords:** liver cancer, China, G20, global burden of disease, joinpoint regression, prediction

## Abstract

**Purpose:**

This study aims to comprehensively compare and analyze the burden of liver cancer in China and the Group of 20 (G20) countries from 1990 to 2021, based on the latest Global Burden of Disease (GBD) 2021 database. It also predicts the trends in liver cancer incidence, prevalence, mortality, and disability-adjusted life years (DALYs) in China and G20 countries over the next 15 years.

**Methods:**

This observational longitudinal study utilizes data from the GBD 2021 database, employing indicators that include incidence, mortality, prevalence, DALYs, and age-standardized rates (ASRs) to assess liver cancer trends. The Joinpoint regression model was used to calculate the annual average percentage change (AAPC) to determine long-term trends of significant changes in liver cancer occurrence in China and G20 countries. The autoregressive integrated moving average (ARIMA) model was employed to predict the burden trends of liver cancer in China and G20 countries from 2022 to 2036.

**Results:**

In 2021, the age-standardized incidence rate (ASIR), the age-standardized prevalence rate (ASPR), age-standardized mortality rate (ASMR), and age-standardized DALYs rate (ASDR) (95% CI) for liver cancer in China were 9.52 (7.72–11.78), 13.29 (10.75–16.41), 8.35 (6.80–10.29) and 239.91 (191.98–299.37) per 100,000 people respectively, indicating a decrease compared to 1990. The ASIR, ASPR, ASMR, and ASDR for G20 countries in 1990 and 2021 were substantially lower than those of China during the same periods. Joinpoint regression analysis demonstrated an overall decline in the ASMR and ASDR of liver cancer in China and G20 countries from 1990 to 2021. Liver cancer incidents and deaths caused by five etiologies also increased from 1990 to 2021, and non-alcoholic steatohepatitis (NASH) was the largest increasing etiologies in China. Infections with hepatitis B virus (HBV) remained the leading etiology of liver cancer in China and G20 countries. Projections indicate that between 2022 and 2036, both China and G20 countries will experience predominantly modest changes in ASRs.

**Conclusion:**

Due to the implementation of preventive strategies, advancements in healthcare, and improved economic conditions, the incidence, prevalence, mortality, and DALYs of liver cancer in China have decreased. However, there remains a certain gap compared to G20 countries at the same time. In the future, China should develop more detailed prevention and control strategies targeting risk factors, tailored to men, women, and different age groups.

## Introduction

Liver cancer is one of the most prevalent malignancies globally and poses a substantial threat to public health worldwide. Ranked as the sixth most common cancer in terms of incidence and the third leading cause of cancer mortality (1), it represents a major global health challenge associated with considerable disease burden, particularly in Asia and China. Approximately 75% of liver cancer cases occur in Asia, with China accounting for over 50% of the global total (2, 3). Research indicates that liver cancer incidence, prevalence, mortality, and disability-adjusted life years (DALYs) in China are significantly higher than global averages. Furthermore, the peak age for the burden of liver cancer in both Chinese men and women occurs earlier compared to other regions (4, 5). Over recent decades, liver cancer rates have declined in numerous countries, especially in former high-burden Asian nations (2, 6). However, incidence is increasing in regions previously characterized by lower rates, such as the United States (7). Diagnosis often occurs at intermediate or advanced stages due to the insidious onset of liver cancer, thereby eliminating the optimal window for curative radical surgery. Even after surgical resection, recurrence or metastasis remains highly probable. Despite recent advancements expanding therapeutic options, including immunotherapy and targeted treatments, the prognosis for patients with unresectable or metastatic disease have not substantially improved. This underscores the critical need to strengthen prevention and control measures for liver cancer.

The Group of 20 (G20), comprising twenty key entities—namely Argentina, Australia, Brazil, Canada, China, France, Germany, India, Indonesia, Italy, Japan, Republic of Korea, Mexico, Russia, Saudi Arabia, South Africa, Turkey, the United Kingdom, the United States, and the European Union (EU) representing its 27 member states (Austria, Belgium, Bulgaria, Croatia, Cyprus, Czechia, Denmark, Estonia, Finland, France, Germany, Greece, Hungary, Ireland, Italy, Latvia, Lithuania, Luxembourg, Malta, Netherlands, Poland, Portugal, Romania, Slovakia, Slovenia, Spain, Sweden)—serves as the principal forum for international economic cooperation. Encompassing a wide range of economies and demonstrating strong representation, the G20 membership achieves a balance of interests by including both developed and developing nations from diverse regions. Its members collectively account for two-thirds of the global population (8). The disease burden within G20 countries exhibits profound interconnections with their respective economic environments and health statuses (9). The Global Burden of Disease (GBD) Study 2021 constitutes a publicly accessible, comprehensive database documenting the burden of over 300 diseases and injuries across 204 countries and territories. Critically, research indicates that the burden of liver cancer varies significantly by geographic location and socioeconomic status (10, 11). While current GBD-based studies on liver cancer burden primarily focus on global trends and specific analyses within individual countries or regions (12–14), comparative analyses have also examined liver cancer trends in China alongside those in Japan and Republic of Korea (15). However, a significant limitation persists: studies directly comparing liver cancer trends between China and a wider range of other nations remain scarce, and exploration of heterogeneity across diverse countries and territories is markedly insufficient. Importantly, this gap impedes China's capacity to identify potential deficiencies within its health policies and hinders opportunities to learn from and adopt effective measures implemented successfully in other countries. Consequently, analyzing both the temporal trends of liver cancer in China and their divergence from trends in other countries is crucial for formulating effective health policies and medical interventions. Therefore, a comparative analysis of disease burden and long-term trends specifically between China and G20 countries holds significant practical relevance; yet, such research is currently absent.

To address this gap, our study leverages data from the GBD 2021 database and employs a suite of rigorous analytical methods to assess the liver cancer burden in China relative to G20 countries. Specifically, key epidemiological indicators—including incidence, prevalence, deaths, and DALYs—were extracted from the GBD database for China and G20 countries. Subsequently, time-trend analysis methods were applied to meticulously characterize the temporal evolution of these indicators over several decades, including the calculation of statistical metrics such as the average annual percentage change (AAPC). Furthermore, employing mathematical modeling, the study projects the likely trajectory of the liver cancer burden over the next 15 years, explicitly integrating the influence of key drivers like demographic shifts and changes in risk factor profiles. Altogether, this study facilitates a comprehensive and systematic comparison of the similarities and differences in liver cancer burden between China and the G20 countries, thereby establishing a robust empirical foundation and scientific basis for subsequent in-depth analyses and the development of targeted prevention and control strategies.

## Methods

### Data source

All data used in this study were extracted from the GBD 2021 database, which was updated on May 16, 2024, by the Institute for Health Metrics and Evaluation (IHME) at the University of Washington, Seattle, USA. The GBD 2021 delivers comprehensive assessments of incidence, prevalence, deaths, DALYs, and 88 risk factors for 371 diseases and injuries across 204 countries and territories, including 811 subnational locations. Since the GBD 2021 data are publicly available and intended solely for academic research, with no commercial use involved, ethical approval for data access and use was not required. The URL information of the data sources: https://vizhub.healthdata.org/gbd-results/. In this tool, we selected “Cause of death or injury” as the “GBD Estimate”; “Liver cancer”, “Liver cancer due to hepatitis B”, “Liver cancer due to hepatitis C”, “Liver cancer due to alcohol use”, “Liver cancer due to other causes”, and “Liver cancer due to NASH” as the “Cause”; “Incidence”, “Prevalence”, “Deaths”, and “DALYs” as the “Measure”; “Number”, “Rate” and “Percent” as the “Metric”; “Global”, “China”, and “G20” as the “Location”; “All ages”, “Age-standardized”, “ < 5 years”, “5–9 years”, “10–14 years”, “15–19 years”, “20–24 years”, “25–29 years”, “30–34 years”, “35–39 years”, “40–44 years”, “45–49 years”, “50–54 years”, “55–59 years”, “60–64 years”, “65–69 years”, “70–74 years”, “75–79 years”, “80–84 years”, “85–89 years”, “90–94 years”, and “95+ years” as the “Age”; “Both”, “Male”, and “Female” as the “Sex”; and select all years from 1990 to 2021 as the “Year”. The G20 aggregate follows IHME's standardized location hierarchy where the EU is treated as a distinct entity. When reporting G20 totals, individual EU member states (e.g., France, Germany) contribute only through the EU aggregate to prevent double-counting. The complete list of G20 constituents includes 19 sovereign nations plus the EU. All estimates were extracted directly from GBD's pre-calculated location-specific results using the GBD results tool.

### Statistical analyses

We selected the incidence, prevalence, deaths, and DALYs of liver cancer in China and G20 countries from the GBD 2021 study, along with the corresponding age-standardized incidence rate (ASIR), age-standardized prevalence rates (ASPR), age-standardized mortality rate (ASMR), and age-standardized DALYs rate (ASDR). The 95% confidence intervals (CIs) were demonstrated for each estimated quantity. All age-standardized rates (ASRs) presented in this study were calculated using the GBD world standard population. This standard population structure is defined and maintained by the Global Burden of Disease Collaborative Network specifically for consistent comparability within GBD studies. The methods for age standardization were consistent across all analyses, including those for China, the G20, and globally. Age and sex-specific data for China and G20 countries were downloaded for the period 1990 to 2021 to assess the liver cancer burden. To validate subgroup trends, we tested sex × year interactions using linear regression. The GBD collaborators classify the etiologies for liver cancer into five groups: hepatitis B (HBV), hepatitis C (HCV), alcohol use, non-alcoholic steatohepatitis (NASH), and other causes. “Cryptogenic” or “idiopathic” liver cancer, liver cancer caused by hemochromatosis, autoimmune hepatitis, and Wilson's disease were included in the “other causes” category. The etiology decomposition analysis illustrated how the population, age, and epidemiological changes contributed to the varying liver cancer burden between 1990 and 2021 (15).

To assess trends in liver cancer burden, the Joinpoint regression analysis was conducted using Joinpoint software (version 5.2.0; Statistical Research and Applications Branch, National Cancer Institute, National Institutes of Health, Bethesda, MD, USA). The Joinpoint regression analysis was employed to identify significant temporal trends in the ASRs. The log-transformed ASRs were modeled as ln(*y*) = α + β*x* + ε, where y represents the ASR and x denotes the calendar year. The maximum number of Joinpoints (inflection points) was set to six to balance model flexibility against overfitting, given the length of the time series. The optimal model was selected using a sequential Monte Carlo permutation test (4,500 repetitions, α = 0.05) rather than information criteria, as this method is established for identifying statistically significant trend changes while favoring more parsimonious models. The model assumed uncorrelated errors, an appropriate choice for annual population-level vital statistics data where serial autocorrelation is typically minimal. To account for the uncertainty in the ASR, a variance weighting scheme based on Poisson distribution was applied. The annual percent change (APC) with its 95% CI was calculated for each identified trend segment (16, 17). The AAPC over the entire study period, a summary measure of the trend, was derived as 100 × [exp(β) – 1], with its 95% CI obtained from the model (18). An increasing trend was defined as an AAPC with a lower 95% CI boundary > 0, while a decreasing trend was indicated by an AAPC with an upper 95% CI boundary < 0. It is important to note that our Joinpoint regression analysis involved testing for trend changes across multiple time segments, indicators (ASIR, ASPR, ASMR, ASDR), and geographical strata (China and G20 countries). This multiplicity of comparisons increases the family-wise error rate, meaning that the probability of falsely identifying a significant trend change (Type I error) across the entire analysis is higher than the significance level (α = 0.05) used for any single test. While *P*-values for each APC segment are presented as per conventional reporting, the interpretation of results prioritizes the point estimates (APCs) and their 95% CIs over isolated statistical significance to provide a more robust assessment of trends.

Then, we used the autoregressive integrated moving average (ARIMA) model to predict the incidence, prevalence, deaths, and DALYs of liver cancer in China and G20 countries from 2022 to 2036. The ARIMA model integrates the autoregressive (AR) and moving average (MA) approaches. Its underlying assumption posits that time series represent time-dependent random variables. ARIMA characterizes their autocorrelation and facilitates predictions of future values based on previously observed values (19). Initially, stationarity testing was performed on all series. For non-stationary series, differencing was applied until stationarity was achieved (*P* < 0.05), with the order of differencing denoted as *d*. Subsequently, based on the differenced stationary series, autocorrelation function (ACF) plot was examined to preliminarily identify the potential range for the autoregressive order *p* and moving average order *q*. The optimal model orders (*p, d, q*) were ultimately determined using the auto.arima() function from the R forecast package, which performs an automated search aimed at minimizing the akaike information criterion (AIC). Model parameters were estimated via maximum likelihood estimation (MLE). Following model establishment, residual diagnostics were conducted to ensure its adequacy: the residual ACF plot was inspected to confirm the absence of autocorrelation structures, and the Ljung-Box test [with the number of lags set to (10)] was performed to verify that the residuals constituted a white noise series (*P* > 0.05). To evaluate the applicability of different time-series forecasting methods to the data in this study, we fitted and compared three commonly used models: (1) the ARIMA model, which excels at capturing the autocorrelation and moving average structure of a series; (2) the ETS (Error, Trend, Seasonality) model, based on exponential smoothing, which is suitable for identifying and forecasting underlying trends, seasonality, and error components in a time series; and (3) the Naive Drift model, which serves as a simple benchmark by considering only the average change between observations. Comparing these models is crucial for identifying the forecasting method that best captures the dynamic characteristics of the specific health indicator. Subsequently, model predictive accuracy on the test set was assessed using metrics such as the root mean square error (RMSE), mean absolute error (MAE), and mean absolute percentage error (MAPE), while the AIC and BIC were employed to evaluate the balance between model goodness-of-fit and complexity.

Data analysis and visualization were performed using R statistical software (version 4.4.1) and Joinpoint software (version 5.2.0). A *P*-value of < 0.05 was considered statistically significant.

## Results

### Characterization of the liver cancer burden in China and G20 countries

In China, the number of liver cancer cases increased from 96,434 (95% CI: 80,971–113,769) in 1990 to 196,637 (95% CI: 158,273–243,558) in 2021. Despite this increase in absolute numbers, the ASIRs experienced a decline from 10.58 per 100,000 people (95% CI: 8.94–12.43) in 1990 to 9.52 per 100,000 people (95% CI: 7.72–11.78) in 2021, resulting in an AAPC of −0.31% (95% CI: −0.39 – −0.23). In G20 countries, liver cancer cases rose from 185,549 (95% CI: 169,557–202,922) to 407,347(95% CI: 365,029–460,802) during the same period, with a slight increase in ASIR from 5.89 per 100,000 people (95% CI: 5.39–6.43) to 6.33 per 100,000 people (95% CI: 5.67–7.16), reflected by an AAPC of 0.20% (95% CI: 0.15–0.24) (Table 1).

**Table 1:** All-ages cases, age-standardized incidence, prevalence, mortality, DALYs, and corresponding AAPC of liver cancer in China and G20 countries in 1990 and 2021.

**Location**	**Measure**	**1990**		**2021**		**1990–2021 AAPC**
		**All-ages cases**	**Age-standardized rates per 100,000 people**	**All-ages cases**	**Age-standardized rates per 100,000 people**	
		***N*** **(95% CI)**	***N*** **(95% CI)**	***N*** **(95% CI)**	***N*** **(95% CI)**	***N*** **(95% CI)**
China	Incidence	96,434 (80,971, 113,769)	10.58 (8.94, 12.43)	196,637 (158,273, 243,558)	9.52 (7.72, 11.78)	−0.31 (−0.39 – −0.23)
	Prevalence	132,779 (108,924, 155,564)	13.51 (11.20, 15.77)	265,539 (212,435, 331,149)	13.29 (10.75, 16.41)	0.02 (−0.11–0.15)
	Deaths	94,937 (79,884, 111,527)	10.75 (9.12, 12.61)	172,068 (139,621, 212496)	8.35 (6.80, 10.29)	−0.68 (−1.25 – −0.10)
	DALYs	3,294,864 (2,763,029, 3,879,589)	334.52 (281.08, 393.14)	4,890,023 (3,905,089, 6,124,599)	239.91 (191.98, 299.37)	−0.96 (−1.36 – −0.56)
G20	Incidence	185,549 (169,557, 202,922)	5.89 (5.39, 6.43)	407,347 (365,029, 460,802)	6.33 (5.67, 7.16)	0.20 (0.15–0.24)
	Prevalence	257,405 (230,105, 282,521)	7.88 (7.11, 8.62)	579,708 (519,502, 652,591)	9.25 (8.30, 10.38)	0.50 (0.46–0.54)
	Deaths	178,992 (163,158, 195,991)	5.75 (5.26, 6.28)	361,134 (323,331, 408,535)	5.59 (5.00, 6.32)	−0.04 (−0.24–0.15)
	DALYs	5,609,295 (5,052,422, 6,184,552)	171.28 (154.62, 188.76)	9,250,469 (8,204,183, 10,595,225)	146.24 (129.45, 167.60)	−0.46 (−0.72 – −0.19)

G20, group of 20; CI, confidence interval; DALYs, disability-adjusted life years; AAPC, average annual percentage change.

In terms of prevalence, the number of liver cancer cases in China more than doubled, increasing from 132,779 (95% CI: 108,924–155,564) in 1990 to 265,539 (95% CI: 212,435–331,149) in 2021. However, the ASPR remained relatively stable, with a slight decrease from 13.51 per 100,000 people (95% CI: 11.20–15.77) to 13.29 per 100,000 people (95% CI: 10.75–16.41), reflected by an AAPC of 0.02% (95% CI: −0.11–0.15). In G20 countries, the ASPR increased from 7.88 per 100,000 people (95% CI: 7.11–8.62) in 1990 to 9.25 per 100,000 people (95% CI: 8.30–10.38) in 2021, reflected by an AAPC of 0.50% (95% CI: 0.46–0.54) (Table 1).

The number of liver cancer deaths in China rose from 94,937 (95% CI: 79,884–111,527) in 1990 to 172,068 (95% CI: 139,621–212,496) in 2021. The ASMR decreased from 10.75 per 100,000 people (95% CI: 9.12–12.61) to 8.35 per 100,000 people (95% CI: 6.80–10.29), with an AAPC of −0.68% (95% CI: −1.25 – −0.10). Meanwhile, the liver cancer deaths in G20 countries increased from 178,992 (95% CI: 163,158–195,991) in 1990 to 361,134 (95% CI: 323,331–408,535) in 2021, while the ASMR remained relatively stable, with a slightly decline from 5.75 per 100,000 people (95% CI: 5.26–6.28) to 5.59 per 100,000 people (95% CI: 5.00–6.32), reflected by an AAPC of −0.04% (95% CI: −0.24–0.15) (Table 1).

The DALY attributed to liver cancer in China escalated from 3,294,864 (95% CI: 2,763,029–3,879,589) in 1990 to 4,890,023 (95% CI: 3,905,089–6,124,599) in 2021. Nonetheless, the ASDR significantly decreased from 334.52 per 100,000 people (95% CI: 281.08–393.14) to 239.91 per 100,000 people (95% CI: 191.98–299.37), resulting in an AAPC of −0.96% (95% CI: −1.36 – −0.56). In G20 countries, DALYs increased from 5,609,295 (95% CI: 5,052,422–6,184,552) in 1990 to 9,250,469 (95% CI: 8,204,183–10,595,225) in 2021, while the ASDR showed a decline from 171.28 per 100,000 people (95% CI: 154.62–188.76) to 146.24 per 100,000 people (95% CI: 129.45–167.60), reflected by an AAPC of −0.46% (95% CI: −0.72 – −0.19) (Table 1).

In China, the number of liver cancer cases increased from 96,434 in 1990 to 196,637 in 2021, representing a 103.91% increase. Among G20 countries, cases rose from 185,549 to 407,347 over the same period, a 119.54% increase. The cumulative number of deaths in China increased by 81.24% between 1990 and 2021, while G20 countries experienced a 191.71% rise. ASIR, ASPR, ASMR and ASDR for liver cancer all declined in China during this period. Conversely, throughout the 1990–2021 period, the G20 countries overall experienced a slight increasing trend in ASIR and ASPR, though ASMR and ASDR decreased. Notably, ASIR, ASPR, ASMR, and ASDR in G20 countries were markedly lower than those in China in both 1990 and 2021.

### Joinpoint regression analysis of liver cancer burden in China and G20 countries

**Figure 1:**
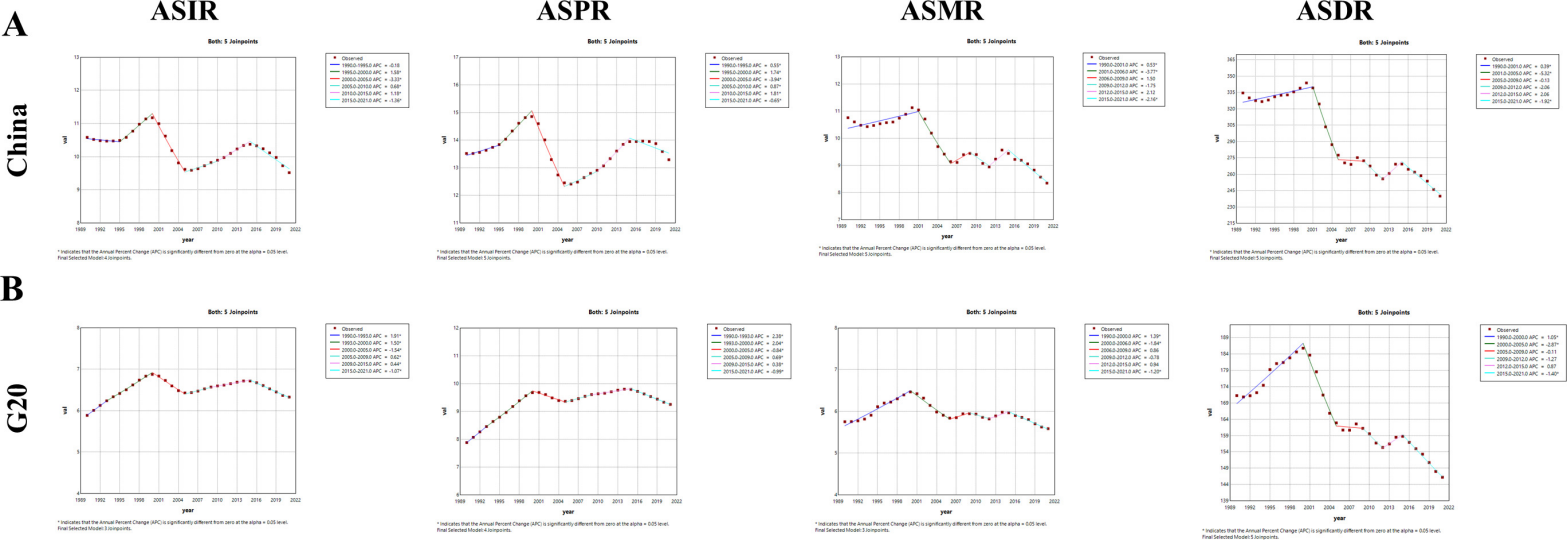
Joinpoint regression analysis of ASIR, ASPR, ASMR, and ASDR of liver cancer in China and G20 countries from 1990 to 2021. **(A)** Joinpoint regression analysis of China's liver cancer ASIR, ASPR, ASMR and ASDR. **(B)** Joinpoint regression analysis of G20 countries' liver cancer ASIR, ASPR, ASMR and ASDR. All analyses are based on raw data without smoothing. * indicates a *P* value < 0.05. G20, Group of 20; ASIR, the age-standardized incidence rate; ASPR, the age-standardized prevalence rate; ASMR, age-standardized mortality rate; ASDR, age-standardized DALYs rate; APC, annual percentage change; AAPC, average annual percentage change.

Joinpoint regression analysis of liver cancer ASIR, ASPR, ASMR, and ASDR trends for China and G20 countries (1990–2021) is presented in Figure 1. The segment-specific APCs are reported in Supplementary 3. In China, ASMR and ASDR exhibited significant increases from 1990 to 2001 (ASMR: APC = 0.53, 95% CI: 0.24 −0.82; ASDR: APC = 0.39, 95% CI: 0.18–0.59). Concurrently during 2000–2005, ASMR and ASDR also demonstrated significant reductions (*P* < 0.05). From 1995 to 2000, ASIR and ASPR for liver cancer in China showed a significant upward trend (ASIR: APC = 1.58, 95% CI: 1.35–1.86; ASPR: APC = 1.74, 95% CI: 1.33–2.16). However, ASIR and ASPR declined significantly between 2000 and 2005 (ASIR: APC = −3.33, 95% CI: −3.60 – −3.19; ASPR: APC = −3.94, 95% CI: −4.26 – −3.62). From 2005 to 2015, ASIR and ASPR displayed renewed increases (*P* < 0.05), whereas ASMR and ASDR trends were non-significant. Finally, all four metrics showed significant declines during 2015–2021 (ASIR: APC = −1.36, 95% CI: −1.99 – −1.40; ASPR: APC = −0.65, 95% CI: −0.98 – −0.33; ASMR: APC = −2.16, 95% CI: −3.12 – −1.20; ASDR: APC = −1.92, 95% CI: −2.63 – −1.20).

In contrast, G20 countries experienced significant increases in all four indicators from 1990 to 2000. Throughout 2000–2021, ASIR and ASPR fluctuated but maintained an overall upward trajectory, while ASMR and ASDR displayed overall declines, with ASDR showing a particularly pronounced downward trend.

### Trends in the burden of liver cancer in China and G20 countries

**Figure 2:**
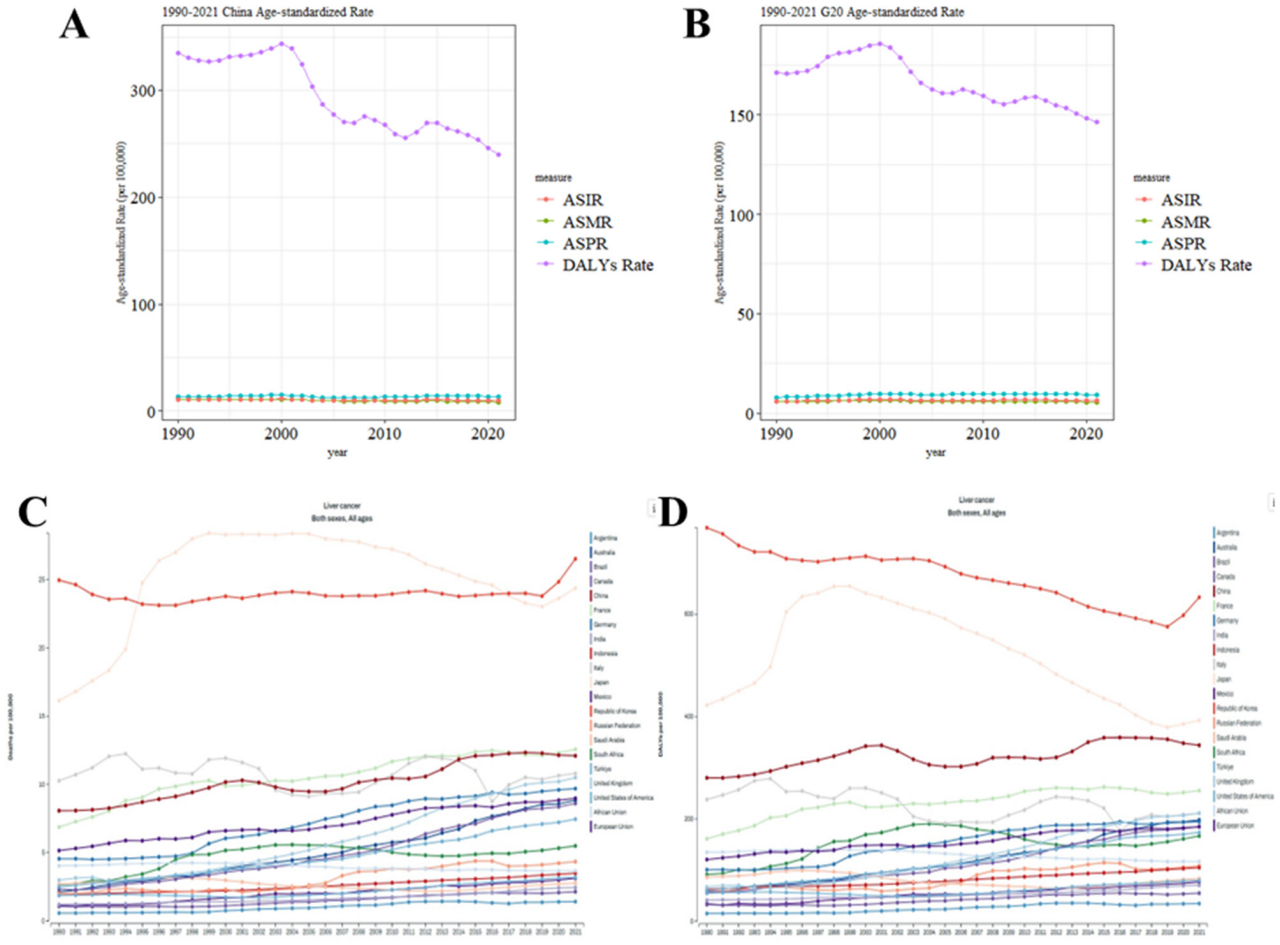
Comparison of the trends in ASIR, ASPR, ASMR, and ASDR of liver cancer in China and G20 countries from 1990 to 2021. **(A)** Comparison of ASIR, ASPR, ASMR, and ASDR trends in China from 1990 to 2021. **(B)** Comparison of ASIR, ASPR, ASMR, and ASDR trends in G20 countries from 1990 to 2021. **(C)** Comparison of ASMR trend in China and other G20 countries from 1990 to 2021. **(D)** Comparison of ASDR trend in China and other G20 countries from 1990 to 2021. All analyses are based on raw data without smoothing. G20, Group of 20; ASIR, the age-standardized incidence rate; ASPR, the age-standardized prevalence rate; ASMR, age-standardized mortality rate; ASDR, age-standardized DALYs rate.

From 1990 to 2021, ASDR of liver cancer in China and G20 countries showed an overall declining trend. Specifically, in China, the ASDR experienced a slight increase from 1990 to 2000, followed by a significant decrease from 2000 to 2005, and then a gradual decline after 2005 (Figure 2A). In contrast, G20 countries exhibited a steady decline in ASDR. Additionally, from 1990 to 2021, both ASIR, ASPR and ASMR of liver cancer in China and G20 countries showed a slight fluctuation trend (Figure 2B).

Figures 2C, D illustrates temporal trends in ASMR and ASDR for liver cancer across all ages and genders in China and other G20 countries (1990–2021). Notably, Japan and South Korea exhibited among the highest ASMR and ASDR rates within the G20. Similarly, China demonstrated persistently elevated rates, with both indicators showing a consistent upward trajectory over the study period.

### Age and sex distribution for liver cancer burden in China and G20 countries

**Figure 3:**
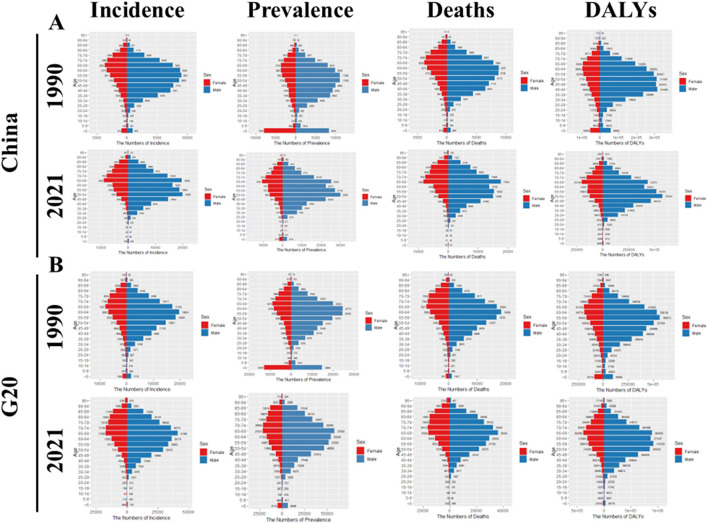
Comparison of the incidence, prevalence, mortality, and DALYs of liver cancer in males and females in different age groups in China and G20 countries in 1990 and 2021. **(A)** Comparison of the incidence, prevalence, mortality, and DALYs of liver cancer in males and females in different age groups in China in 1990 and 2021. **(B)** Comparison of the incidence, prevalence, mortality, and DALYs of liver cancer in males and females in different age groups in G20 countries in 1990 and 2021. All analyses are based on raw data without smoothing. G20, Group of 20; DALYs, disability-adjusted life years.

**Figure 4:**
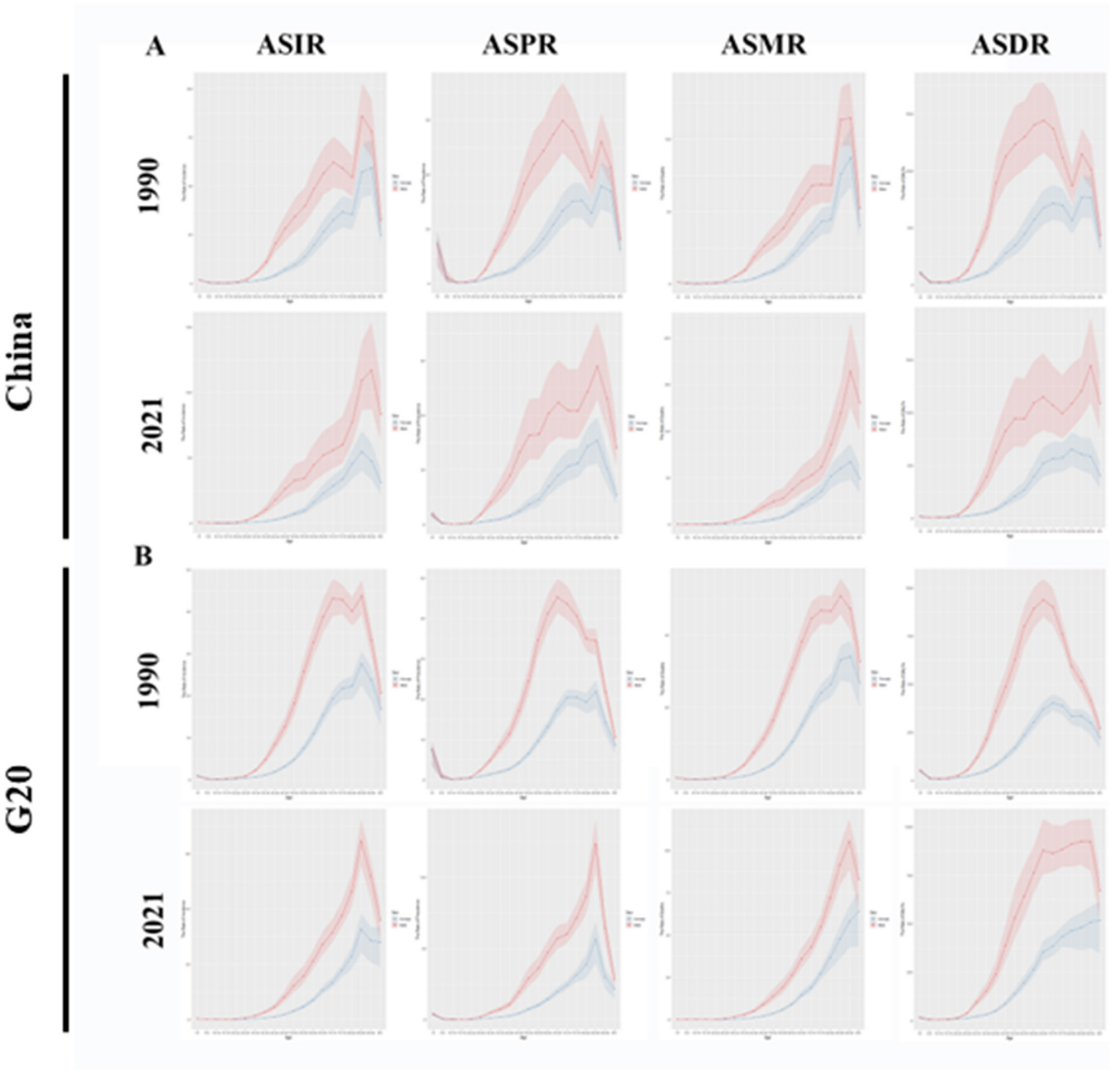
Comparison of ASIR, ASPR, ASMR, and ASDR for different age groups in China and G20 countries in 1990 and 2021. **(A)** ASIR, ASPR, ASMR, and ASDR for different age groups in China in 1990 and 2021. **(B)** ASIR, ASPR, ASMR, and ASDR for different age groups in G20 countries in 1990 and 2021. All analyses are based on raw data without smoothing. G20, Group of 20; ASIR, the age-standardized incidence rate; ASPR, the age-standardized prevalence rate; ASMR, age-standardized mortality rate; ASDR, age-standardized DALYs rate.

Figures 3, 4 display the incidence, prevalence, deaths, and DALYs numbers for liver cancer across different age groups in China and G20 countries from 1990 to 2021 (Figure 3) and the ASRs (Figure 4). Liver cancer is more prevalent in individuals aged over 35 in both China and G20 countries, showing substantial increases between ages 35–85. Age-specific analyses reveal these temporal patterns: For ASIR, peak incidence in 1990 occurred at 85–89 years for males (China, G20) and 90–94 years for Chinese females vs. 85–89 years for G20 females; by 2021, Chinese females peaked earlier at 85–89 years (previously 90–94 in 1990) while Chinese males peaked later at 90–94 years, with G20 males and females maintaining peaks at 85–89 years. For ASPR, 1990 peaks were at 65–69 years for males (China, G20) and 85–89 years for females, shifting to 85–89 years for all groups by 2021. Regarding ASMR, China maintained peaks at 90–94 years for both sexes (1990/2021), while G20 shifted from male (85–89 years) and female (90–94 years) peaks in 1990 to delayed 2021 peaks at 90–94 years (males) and >95 years (females). For ASDR, Chinese males shifted from 65–69 years (1990) to 90–94 years (2021), while females peaked earlier (80–84 years in 2021 vs. 85–89 years in 1990). G20 males shifted from 65–69 years to 90–94 years, with females peaking later (>95 years in 2021 vs. 70–74 years in 1990). Notably, in both 1990 and 2021, the incidence, prevalence, deaths, and DALYs rates for males in China and G20 countries were higher than those for females. It is also noteworthy that the peak incidence and DALYs rate of liver cancer in Chinese women are both advanced.

**Figure 5:**
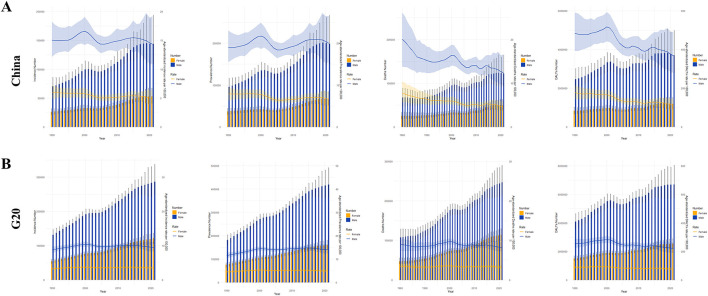
Comparison of full-age cases and age-standardized rates of incidence, prevalence, mortality and DALYs among men and women in China and G20 countries from 1990 to 2021. **(A)** Number of cases and ASIR, ASPR, ASMR, and ASDR for males and females of all ages in China. **(B)** Number of cases and ASIR, ASPR, ASMR, and ASDR for males and females of all ages in G20 countries. The bar graphs represent counts, while the lines represent age-standardized rates. All analyses are based on raw data without smoothing. G20, Group of 20; ASIR, the age-standardized incidence rate; ASPR, the age-standardized prevalence rate; ASMR, age-standardized mortality rate; ASDR, age-standardized DALYs rate.

Figure 5 illustrates the all-age case counts and ASR (incidence, prevalence, mortality, and DALYs) by gender for liver cancer in China and G20 countries from 1990 to 2021. Gender-specific and ASR of incidence, prevalence, mortality, and DALYs exhibited year-to-year fluctuations. Overall, China experienced declining trends in ASMR and ASDR, while ASIR and ASPR showed stable fluctuations. Conversely, all-age case counts of incidence, prevalence, mortality, and DALYs increased consistently. Among G20 countries, ASIR, ASPR, ASMR, and ASDR demonstrated broadly fluctuating patterns, yet all-age case counts trended upward. Notably, males consistently displayed higher ASIR, ASPR, ASMR, ASDR, and all-age case counts than females across all observations.

Based on the data from the sex × year interaction analysis in China and G20 countries (Supplementary 4), the results demonstrate highly significant overall models for all age-standardized metrics (ASIR, ASPR, ASMR, ASDR) across both groups, evidenced by exceptional model fits (adjusted *R*^2^ > 0.99) and robust overall significances (all *F*-statistics *P* < 0.001). Specifically, in China, significant sex-specific temporal interactions (*P* < 0.05) were detected for ASPR, ASMR, and ASDR, revealing diverging gender trends: ASPR exhibited a female decline (−0.04/year) contrasted with a male increase (+0.03/year), while ASMR and ASDR showed reductions for both sexes, albeit steeper for males (ASMR male: −0.09/year vs. female: −0.05/year; ASDR male: −4.44/year vs. female: −2.20/year). For G20 countries, significant interactions emerged for ASIR, ASMR, and ASDR, with both genders displaying slight rises in ASIR and ASPR (ASIR: male: +0.01/year; female: +0.00/year; ASPR male: +0.07/year; female: +0.01/year) but declines in ASMR and ASDR, where males experienced larger decreases (ASMR: male: −0.02/year vs. female: −0.00/year; ASDR male: −1.70/year vs. female: −0.53/year). Non-significant interactions for ASIR in both groups (*P* > 0.05) suggested similar, albeit mild, trends by gender, such as gradual female and male reductions in China vs. slight increases in G20. These findings underscore distinct dynamics of ASIR, ASPR, ASMR, ASDR, with China's patterns indicating greater gender divergence in certain indicators compared to G20′s more uniform trends, highlighting contextual influences on sex-specific changes.

### Cause distribution of liver cancer burden in China, and G20 countries

**Figure 6:**
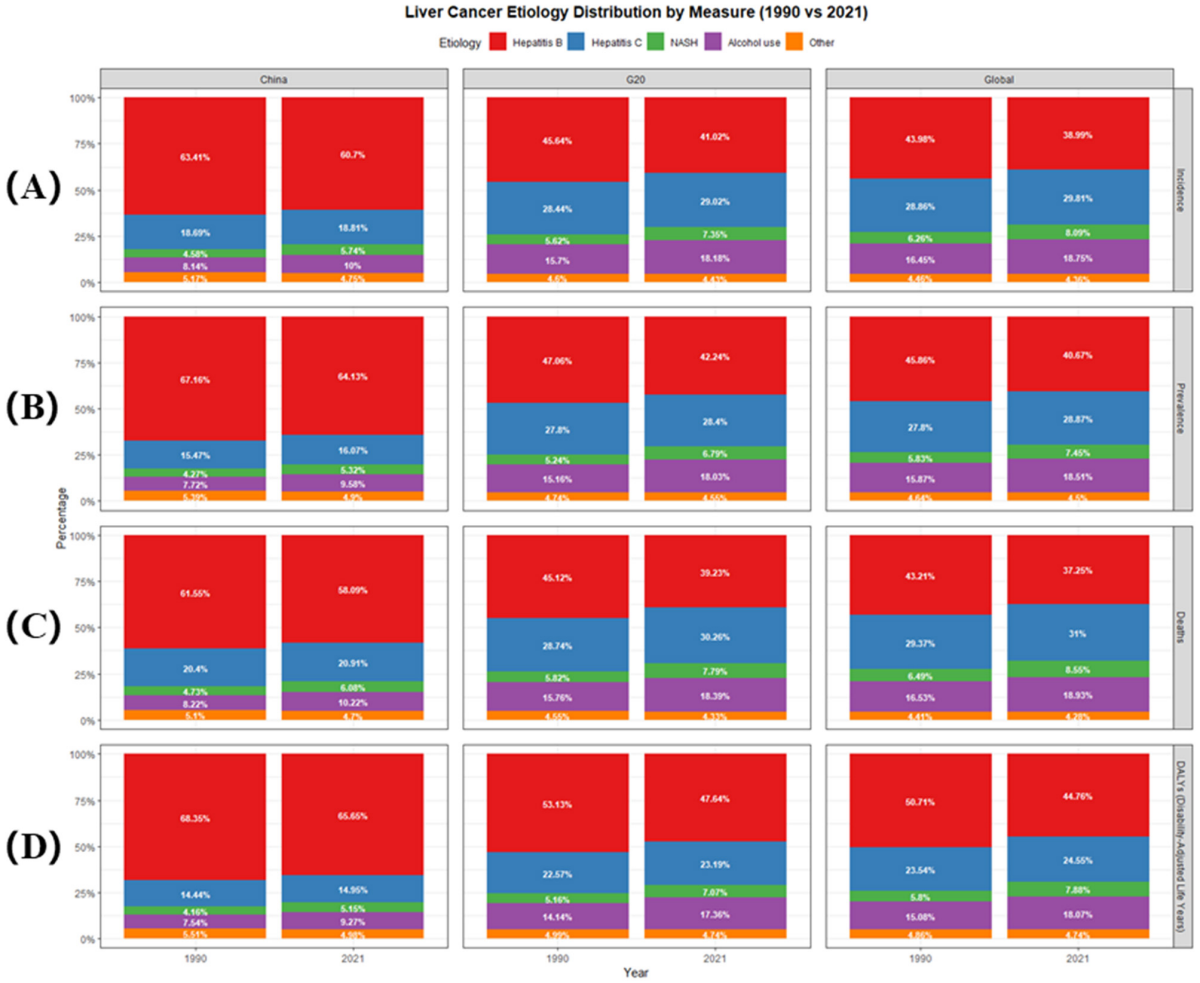
Cause distribution of liver cancer burden in China, and G20 in 1990 and 2021. **(A)** Incidence; **(B)** Prevalence; **(C)** Deaths; **(D)** DALYs. G20, Group of 20; DALYs, disability-adjusted life years; NASH, non-alcoholic steatohepatitis.

Tables 2, 3 summarize the ASRs and case numbers for five etiologies of liver cancer in China and G20 countries for 1990 and 2021, with temporal trends from 1990 to 2021 illustrated in Figure 6. During this period, the case number of incidence, prevalence, deaths, and DALYs attributable to these five etiologies increased in China and G20 countries. In China, NASH exhibited the most substantial growth in incident cases (+178.36%), prevalence (+203.36%), deaths (+152.16%), and DALYs (+104.72%). This pattern of NASH being the most rapidly increasing cause of liver cancer was also consistent in G20 countries and worldwide. Between 1990 and 2021, China experienced a decline in the ASIR for liver cancer related to HBV and HCV, while the ASIRs for NASH and alcohol-related liver cancer rose. The etiological profile of liver cancer in China was generally similar to that in G20 countries. At the global level in 2021, HBV infection was the predominant cause, accounting for 38.99% of incident liver cancer cases, followed by HCV (29.81%), alcohol use (18.75%), NASH (8.09%), and other causes (4.36%). A comparable distribution was observed for mortality, with HBV responsible for 37.25% of deaths, HCV for 31.00%, alcohol use for 18.93%, NASH for 8.55%, and other causes for 4.28% (Figure 6). HBV was the leading etiology in both China and G20 countries, contributing to 60.70% of incident cases and 58.09% of deaths in China, and 41.02% of cases and 39.23% of deaths in the G20 group. HCV was the second leading contributor to the liver cancer burden in these populations.

**Table 2:** The ASR and number of five etiologies of liver cancer in the world, China, and the G20 in 1990, both sexes.

**Location**	**Etiologies**	**Incidence**		**Prevalence**		**Deaths**		**DALYs**	
		**Numbers**	**ASR per 100,000 people (95% CI)**	**Numbers**	**ASR per 100,000 people (95% CI)**	**Numbers**	**ASR per 100,000 people (95% CI)**	**Numbers**	**ASR per 100,000 people (95% CI)**
China	Hepatitis B	63,118 (52,018, 75,227)	6.58 (5.45, 7.84)	80,153 (65,848, 95,282)	7.97 (6.55, 9.46)	61,415 (50,743, 73,122)	6.53 (5.42, 7.76)	2,236,077 (1,842,616, 2,663,358)	220.05 (181.34, 260.91)
	Hepatitis C	14,422 (11,688, 17,539)	1.94 (1.57, 2.32)	14,897 (11,923, 18,145)	1.84 (1.49, 2.23)	15,268 (12,408, 18,500)	2.16 (1.77, 2.60)	386,481 (309,229, 471,854)	46.48 (37.56, 56.75)
	NASH	4,057 (3,237, 4,978)	0.48 (0.38, 0.58)	4,637 (3,727, 5,699)	0.51 (0.41, 0.62)	4,128 (3,293, 5,068)	0.50 (0.40, 0.61)	125,153 (100,593, 153,269)	13.39 (10.77, 16.4)
	Alcohol use	7,500 (5,772, 9,563)	0.84 (0.66, 1.07)	8,484 (6,509, 10,955)	0.92 (0.71, 1.17)	7,575 (5,858, 9,677)	0.87 (0.69, 1.10)	227,509 (174,534, 293,034)	24.26 (18.72, 31.11)
	Other	5,068 (4,017, 6,305)	0.54 (0.43, 0.66)	6,413 (5,111, 7,986)	0.64 (0.51, 0.79)	4,981 (3,957, 6,185)	0.54 (0.43, 0.67)	180,928 (145,486, 225,056)	17.74 (14.16, 21.98)
G20	Hepatitis B	86,219 (74,548, 99,187)	2.64 (2.28, 3.04)	110,485 (95,981, 126,804)	3.32 (2.88, 3.80)	83,179 (71,808, 95,879)	2.56 (2.21, 2.96)	2,941,870 (2,540,864, 3,379,475)	87.74 (75.79, 101.08)
	Hepatitis C	48,869 (43,496, 54,420)	1.64 (1.47, 1.82)	59,878 (53,770, 66,406)	1.96 (1.76, 2.16)	47,575 (42,302, 53,153)	1.63 (1.45, 1.82)	1,150,436 (1,017,345, 1,295,476)	37.28 (33.11, 41.96)
	NASH	9,934 (8,261, 11,653)	0.32 (0.27, 0.38)	11,624 (9,809, 13,646)	0.37 (0.31, 0.43)	9,950 (8,292, 11,761)	0.33 (0.27, 0.39)	271,992 (229,892, 320,023)	8.52 (7.17, 10.01)
	Alcohol use	28,175 (24,017, 32,784)	0.91 (0.77, 1.06)	33,720 (28,780, 39,201)	1.07 (0.91, 1.24)	27,515 (23,382, 32,128)	0.90 (0.76, 1.05)	742,945 (633,134, 873,905)	23.36 (19.85, 27.43)
	Other	8,528 (7,141, 10,267)	0.27 (0.22, 0.32)	11,020 (9,275, 13,222)	0.33 (0.28, 0.40)	8,210 (6,872, 9,854)	0.26 (0.22, 0.31)	275,988 (231,001, 331,117)	8.25 (6.91, 9.89)
World	Hepatitis B	109,479 (94,820, 127,366)	2.55 (2.2, 2.97)	138,790 (121,366, 160,866)	3.14 (2.74, 3.64)	106,514 (91,940, 124,291)	2.50 (2.15, 2.92)	3,748,179 (3,255,634, 4,362,511)	84.16 (73.10, 97.96)
	Hepatitis C	64,373 (55,670, 75,392)	1.67 (1.45, 1.95)	75,728 (66,439, 87306)	1.91 (1.67, 2.19)	64,130 (55,297, 75,525)	1.70 (1.47, 1.99)	1,572,206 (1,347,332, 1,870,515)	39.07 (33.57, 46.31)
	NASH	14,414 (11,471, 17,854)	0.36 (0.29, 0.45)	16,454 (13,195, 20,257)	0.40 (0.32, 0.49)	14,675 (11,621, 18,159)	0.38 (0.30, 0.47)	404,013 (321,351, 499,991)	9.63 (7.66, 11.90)
	Alcohol use	38,445 (31,540, 46,399)	0.95 (0.78, 1.14)	44,848 (37,055, 54,444)	1.09 (0.90, 1.31)	38,172 (31,170, 46,200)	0.96 (0.78, 1.15)	1,042,116 (852,871, 1,280,544)	25.03 (20.59, 30.50)
	Other	10,915 (8,927, 13,584)	0.26 (0.21, 0.32)	13,910 (11,558, 17,065)	0.32 (0.26, 0.39)	10,650 (8,701, 13,326)	0.26 (0.21, 0.32)	360,789 (299,263, 444,146)	8.07 (6.64, 9.97)

G20, group of 20; NASH, non-alcoholic steatohepatitis; DALYs, disability-adjusted life years; ASR, age-standardized rate; CI, confidence interval.

**Table 3:** The ASR and number of five etiologies of liver cancer in the world, China, and the G20 in 2021, both sexes.

**Location**	**Etiologies**	**Incidence**		**Prevalence**		**Deaths**		**DALYs**	
		**Numbers**	**ASR per 100,000 people (95% CI)**	**Numbers**	**ASR per 100,000 people (95% CI)**	**Numbers**	**ASR per 100,000 people (95% CI)**	**Numbers**	**ASR per 100,000 people (95% CI)**
**China**	Hepatitis B	118,665 (92,280, 153,556)	5.73 (4.48, 7.38)	165,217 (129,037, 215,080)	8.08 (6.33, 10.47)	100,194 (77,721, 129,138)	4.83 (3.76, 6.19)	3,148,553 (2,442,865, 4,109,014)	155.81 (121.32,201.99)
	Hepatitis C	36,427 (28,404, 44,840)	1.78 (1.41, 2.18)	42,632 (33,088, 52,910)	2.02 (1.58, 2.50)	34,899 (27,413, 42,965)	1.74 (1.38, 2.12)	751,020 (585,482, 933,695)	35.49 (27.67,44.05)
	NASH	11,293 (8,663, 14,314)	0.54 (0.42, 0.68)	14,067 (10,634, 17,931)	0.67 (0.51, 0.85)	10,409 (8,036, 13,180)	0.51 (0.39, 0.64)	256,209 (194,368, 326,023)	12.22 (9.41,15.44)
	Alcohol use	20,464 (15,239, 27,296)	0.94 (0.71, 1.25)	26,242 (19,417, 34,985)	1.21 (0.90, 1.59)	18,317 (13,653, 24,252)	0.85 (0.64, 1.12)	477,847 (352,518, 637,755)	22.01 (16.3, 29.15)
	Other	9,235 (7,034, 11,875)	0.45 (0.34, 0.57)	12,551 (9,635, 16,318)	0.62 (0.48, 0.80)	8,033 (6,102, 10,231)	0.39 (0.30, 0.49)	237,416 (180,748, 310,459)	11.82 (9.11,15.35)
**G20**	Hepatitis B	163,605 (132,910, 201,275)	2.57 (2.10, 3.17)	233,895 (190,628, 287,003)	3.72 (3.04,4.56)	139,435 (113,354, 170,864)	2.18 (1.78, 2.66)	4,270,828 (3,475,990, 5,282,465)	68.71 (56.23, 84.80)
	Hepatitis C	119,052 (101,927, 135,306)	1.82 (1.56, 2.07)	163,894 (141,168, 185,730)	2.50 (2.16, 2.84)	109,694 (93,983, 124,573)	1.68 (1.44, 1.91)	2,192,140 (1,890,901, 2,521,669)	33.44 (28.81, 38.44)
	NASH	30,050 (24,392, 35935)	0.46 (0.38, 0.55)	38,760 (32,090, 46,217)	0.60 (0.50, 0.71)	28,221 (23,167, 33,830)	0.43 (0.36, 0.52)	655,980 (540,885, 779,131)	10.19 (8.45, 12.04)
	Alcohol use	75,338 (63,237, 88,922)	1.14 (0.96, 1.35)	104,583 (87,501, 122,560)	1.59 (1.34, 1.86)	67,616 (56,751, 79,588)	1.02 (0.86, 1.20)	1,642,214 (1,382,202, 1,948,474)	25.04 (21.13,29.71)
	Other	17,766 (14,279, 21,510)	0.28 (0.22, 0.33)	25,329 (20,614, 30,509)	0.40 (0.33, 0.48)	15,455 (12,363, 18,559)	0.24 (0.19, 0.29)	426,289 (347,723, 524,495)	6.84 (5.62, 8.39)
**World**	Hepatitis B	206,366 (169,401, 252,050)	2.37 (1.95, 2.89)	288,106 (237,812, 349,750)	3.32 (2.74, 4.02)	181,194 (148,896, 221,685)	2.09 (1.72, 2.55)	5,668,199 (4,706,886, 6,885,071)	65.36 (54.43, 79.35)
	Hepatitis C	154,062 (131,916, 177,255)	1.82 (1.56, 2.08)	201,212 (173,233, 228,763)	2.36 (2.03, 2.68)	146,522 (125,936, 168,519)	1.74 (1.49, 1.99)	3,098,870 (2,662,298, 3,609,082)	35.85 (30.84, 41.7)
	NASH	42,291 (34,033, 51,129)	0.49 (0.40, 0.60)	52,431 (42,488, 63,205)	0.61 (0.49, 0.73)	40925 (32,961, 49,610)	0.48 (0.39, 0.58)	995,475 (808,799, 1,201,789)	11.50 (9.39,13.84)
	Alcohol use	99,544 (80,957, 120,402)	1.14 (0.93, 1.38)	132,033 (107,264, 159,049)	1.51 (1.23, 1.82)	92,228 (75,053, 112,160)	1.06 (0.86, 1.29)	2,316,027 (1,887,013, 2,845,789)	26.39 (21.53, 32.28)
	Other	22,892 (18,375, 27,801)	0.27 (0.21, 0.32)	31,688 (25,737, 38,475)	0.37 (0.30, 0.44)	20,590 (16,371, 25,048)	0.24 (0.19, 0.29)	595,603 (484,797, 732,313)	6.92 (5.64, 8.48)

G20, group of 20; NASH, non-alcoholic steatohepatitis; DALYs, disability-adjusted life years; ASR, age-standardized rate; CI, confidence interval.

### Prediction of incidence, prevalence, mortality and DALYs of liver cancer in China and G20 countries during 2022–2036

We fitted the ASIR, ASPR, ASMR, and ASDR of liver cancer stratified by gender from 1990 to 2021 using the ARIMA model and predicted the ASIR, ASPR, ASMR, and ASDR for 2022–2036, selecting the optimal model parameters along with the corresponding AIC and BIC (Supplementary 1). Supplementary 1 detail the performance metrics (RMSE, MAE, and MAPE) of the ARIMA, ETS, and Naive Drift models in forecasting ASIR, ASPR, ASMR, and ASDR for both male and female in China and the G20. The ACF test diagram of ARIMA model and the prediction trend diagram of ETS and naive drift model are summarized in Supplementary 2. The results demonstrate that the ARIMA model achieved the best predictive accuracy (lowest RMSE, MAE, and MAPE) for the majority of indicators, particularly for ASIR and ASPR, which further validates its selection for this analysis.

**Figure 7:**
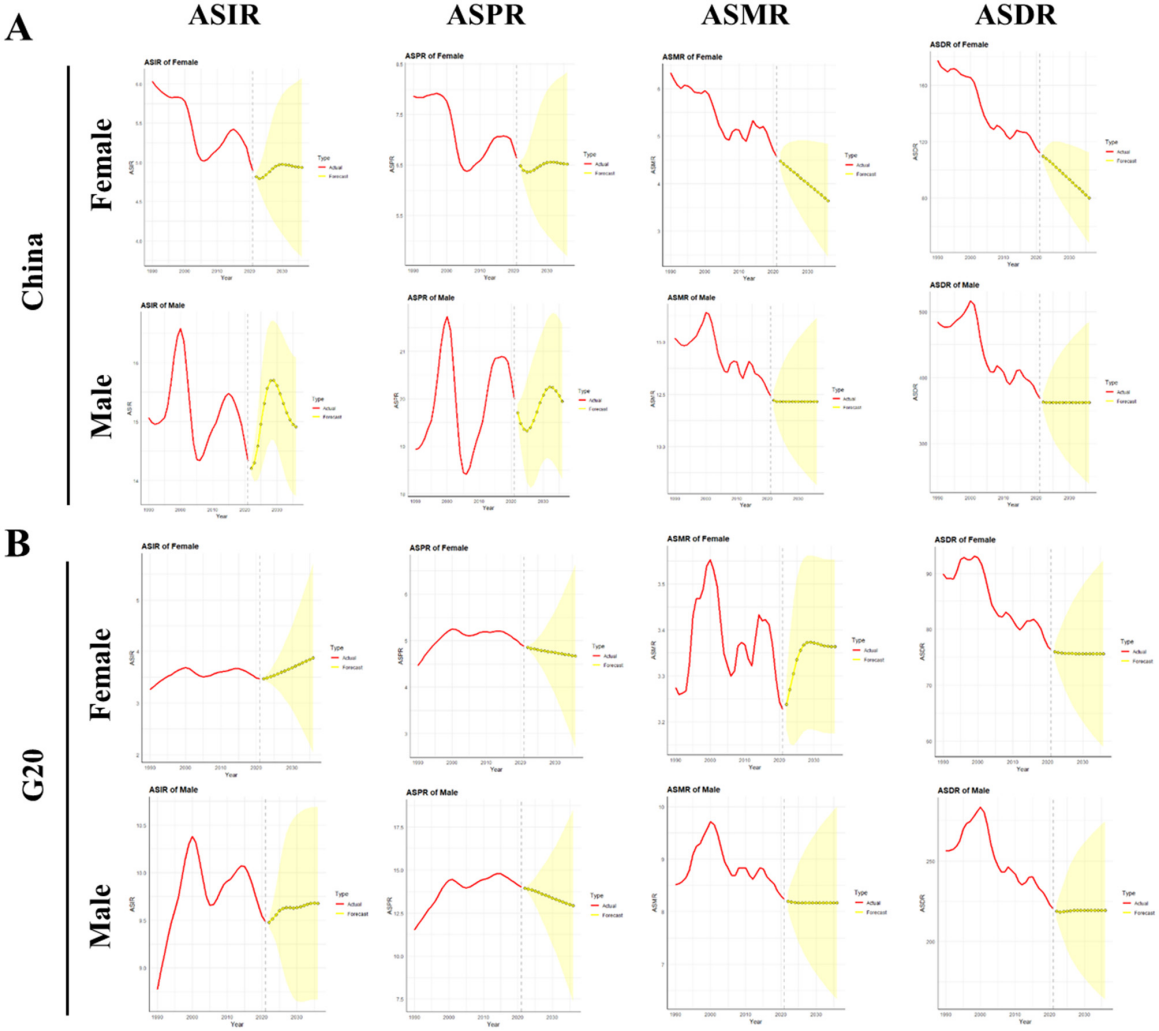
Temporal Trends of ASIR, ASPR, ASMR, and ASDR of Liver Cancer in China and G20 Countries from 1990 to 2021, with Projections for 2022–2036. **(A)** Temporal trends of ASIR, ASPR, ASMR, and ASDR of liver cancer in China from 1990 to 2021, along with projections for 2022-2036. **(B)** Temporal trends of ASIR, ASPR, ASMR, and ASDR of liver cancer in G20 countries from 1990 to 2021, along with projections for 2022–2036. The red solid line represents the observed data. The yellow dashed line shows the fitted values from the ARIMA model. The yellow solid line extending beyond the vertical marker indicates the forecast, with the shaded area representing the 95% confidence interval. All analyses are based on raw data without smoothing. G20, Group of 20; ASIR, the age-standardized incidence rate; ASPR, the age-standardized prevalence rate; ASMR, age-standardized mortality rate; ASDR, age-standardized DALYs rate.

Between 2022 and 2036, Chinese male ASIR is projected to increase from 14.21 to 14.91 per 100,000 people, representing a 4.93% rise. The female ASIR will climb from 4.82 to 4.93 per 100,000 people, marking a 2.28% increase. In contrast, G20 countries will see their male ASIR rise from 9.48 to 9.68 per 100,000 people (2.11% increase), while the female ASIR will grow from 3.47 to 3.87 per 100,000 people, corresponding to a 11.53% increase. ASPR for males in China is forecasted to increase slightly from 19.71 to 19.95 per 100,000 people (1.22% increase), with the female ASPR rising minimally from 6.49 to 6.52 per 100,000 people (0.46% increase). Conversely, G20 countries are expected to experience a male ASPR decline from 13.94 to 12.92 per 100,000 people (7.32% decrease), while the female ASPR will decrease marginally from 8.20 to 8.17 per 100,000 people (0.36% decrease). The male ASMR in China is predicted to decline slightly from 12.22 to 12.16 per 100,000 people (0.49% decrease), whereas the female ASMR will decrease substantially from 4.48 to 3.64 per 100,000 people (18.75% decline). In G20 countries, male ASMR will decrease from 8.20 to 8.17 per 100,000 people (0.37% decrease), but female ASMR will increase from 3.24 to 3.36 per 100,000 people (3.70% rise). Regarding ASDR, Chinese males will see a marginal reduction from 363.10 to 362.11 per 100,000 people (0.27% decrease), while males in G20 countries will experience a slight increase from 218.74 to 219.35 per 100,000 people (0.28% increase). Chinese females will demonstrate a significant ASDR reduction from 109.70 to 80.15 per 100,000 people (26.94% decline), compared to a minor decrease among females in G20 countries from 75.98 to 75.64 per 100,000 people (0.45% decrease). Overall, model projections indicate that between 2022 and 2036, both China and G20 countries will experience predominantly modest changes in ASRs (Table 4 and Figure 7). For China, the majority of projected changes fall within a narrow range: ASIR increases by 4.93% (male) and 2.28% (female), ASPR increases by 1.22% (male) and 0.46% (female), ASMR decreases by 0.49% (male) and increases by 3.70% (female), and ASDR decreases by 0.27% (male). Notably, Chinese females show a substantial ASMR decrease (18.75%) and ASDR decrease (26.94%). In G20 countries, changes are similarly contained for most metrics: ASIR increases by 2.11% (male) and 11.53% (female), ASPR decreases by 7.32% (male) and 0.36% (female), ASMR decreases by 0.37% (male) and increases by 3.70% (female), and ASDR increases by 0.28% (male) and decreases by 0.45% (female). The most significant deviations from this pattern of limited change are the larger increase in female ASIR in G20 countries and the pronounced decreases in female ASMR and ASDR in China.

**Table 4:** Projections of ASIR, ASPR, ASMR and ASDR for liver cancer in China and G20 countries from 2022 to 2036.

**Location**	**Year**	**Age-standardized incidence rate, per 100,000 people (95% CI)**		**Age-standardized prevalence rate, per 100,000 people (95% CI)**		**Age-standardized mortality rate, per 100,000 (95% CI)**		**Age-standardized DALYs rate, per 100,000 (95% CI)**	
		**Male**	**Female**	**Male**	**Female**	**Male**	**Female**	**Male**	**Female**
China	2022	14.21 (14.10, 14.32)	4.82 (4.76, 4.88)	19.71 (19.53, 19.90)	6.49 (6.39, 6.58)	12.22 (11.74, 12.69)	4.48 (4.29, 4.67)	363.10 (350.38, 375.83)	109.70 (105.74, 113.66)
	2023	14.30 (13.99, 14.62)	4.79 (4.64, 4.95)	19.49 (18.98, 20.00)	6.40 (6.15, 6.64)	12.16 (11.14, 13.17)	4.42 (4.05, 4.79)	362.11 (333.30, 390.93)	107.89 (99.32, 116.47)
	2024	14.59 (14.01, 15.16)	4.81 (4.53, 5.08)	19.36 (18.52, 20.19)	6.36 (5.91, 6.80)	12.16 (10.68, 13.63)	4.36 (3.88, 4.84)	362.11 (318.21, 406.02)	106.08 (93.35, 118.80)
	2025	14.96 (14.16, 15.76)	4.84 (4.43, 5.25)	19.33 (18.23, 20.43)	6.37 (5.71, 7.02)	12.16 (10.33, 13.98)	4.30 (3.72, 4.88)	362.11 (307.11, 417.12)	104.09 (88.13, 120.04)
	2026	15.31 (14.36, 16.26)	4.88 (4.35, 5.42)	19.40 (18.13, 20.67)	6.40 (5.54, 7.26)	12.16 (10.03, 14.28)	4.24 (3.58, 4.90)	362.11 (297.91, 426.32)	101.95 (83.56, 120.33)
	2027	15.57 (14.56, 16.57)	4.92 (4.27, 5.57)	19.54 (18.19, 20.90)	6.45 (5.41, 7.49)	12.16 (9.78, 14.54)	4.18 (3.45, 4.91)	362.11 (289.86, 434.36)	99.73 (79.44, 120.03)
	2028	15.70 (14.69, 16.71)	4.95 (4.20, 5.70)	19.72 (18.35, 21.10)	6.49 (5.29, 7.69)	12.16 (9.54, 14.77)	4.12 (3.33, 4.91)	362.11 (282.63, 441.60)	97.51 (75.60, 119.43)
	2029	15.70 (14.69, 16.72)	4.97 (4.14, 5.79)	19.92 (18.54, 21.29)	6.53 (5.20, 7.85)	12.16 (9.33, 14.98)	4.06 (3.21, 4.91)	362.11 (276.00, 448.23)	95.32 (71.91, 118.72)
	2030	15.61 (14.57, 16.66)	4.97 (4.08, 5.87)	20.08 (18.69, 21.47)	6.55 (5.12, 7.97)	12.16 (9.13, 15.18)	4.00 (3.09, 4.91)	362.11 (269.85, 454.38	93.14 (68.32, 117.96)
	2031	15.47 (14.38, 16.56)	4.97 (4.03, 5.92)	20.20 (18.76, 21.64)	6.56 (5.04, 8.07)	12.16 (8.94, 15.37)	3.94 (2.98, 4.90)	362.11 (264.08, 460.14)	90.98 (64.79, 117.16)
	2032	15.31 (14.18, 16.44)	4.96 (3.97, 5.95)	20.25 (18.75, 21.76)	6.55 (4.97, 8.14)	12.16 (8.77, 15.54)	3.88 (2.87, 4.89)	362.11 (258.63, 465.59)	88.82 (61.31, 116.32)
	2033	15.16 (14.00, 16.32)	4.95 (3.92, 5.99)	20.24 (18.67, 21.81)	6.55 (4.90, 8.20)	12.16 (8.60, 15.71)	3.82 (2.76, 4.88)	362.11 (253.46, 470.77)	86.65 (57.89, 115.41)
	2034	15.03 (13.86, 16.21)	4.94 (3.87, 6.01)	20.17 (18.56, 21.79)	6.54 (4.83, 8.24)	12.16 (8.44, 15.87)	3.76 (2.66, 4.87)	362.11 (248.53, 475.70)	84.49 (54.52, 114.45)
	2035	14.95 (13.77, 16.13)	4.94 (3.83, 6.04)	20.07 (18.44, 21.70)	6.52 (4.76, 8.29)	12.16 (8.29, 16.02)	3.70 (2.55, 4.85)	362.11 (243.79, 480.44)	82.32 (51.20, 113.44)
	2036	14.91 (13.74, 16.09)	4.93 (3.79, 6.08)	19.95 (18.31, 21.58)	6.52 (4.69, 8.34)	12.16 (8.14, 16.17)	3.64 (2.45, 4.83)	362.11 (239.24, 484.99)	80.15 (47.92, 112.38)
G20	2022	9.48 (9.43, 9.52)	3.47 (3.45, 3.5)	13.94 (13.87, 14.01)	4.85 (4.82, 4.89)	8.20 (8.02, 8.38)	3.24 (3.18, 3.29)	218.74 (213.79, 223.69)	75.98 (74.60, 77.35)
	2023	9.51 (9.36, 9.66)	3.49 (3.42, 3.56)	13.88 (13.65, 14.12)	4.83 (4.75, 4.92)	8.19 (7.80, 8.58)	3.27 (3.15, 3.39)	218.37 (206.98, 229.76)	75.82 (72.55, 79.08)
	2024	9.56 (9.26, 9.86)	3.51 (3.37, 3.65)	13.83 (13.34, 14.32)	4.82 (4.66, 4.98)	8.18 (7.60, 8.76)	3.30 (3.15, 3.46)	218.55 (200.57, 236.54)	75.73 (70.72, 80.74)
	2025	9.60 (9.12, 10.08)	3.54 (3.32, 3.75)	13.77 (12.96, 14.58)	4.80 (4.55, 5.06)	8.17 (7.43, 8.92)	3.33 (3.16, 3.51)	218.89 (195.02, 242.77)	75.69 (69.14, 82.24)
	2026	9.63 (8.98, 10.27)	3.57 (3.25, 3.88)	13.69 (12.53, 14.86)	4.79 (4.43, 5.15)	8.17 (7.28, 9.06)	3.36 (3.17, 3.54)	219.19 (190.39, 247.99)	75.66 (67.75, 83.58)
	2027	9.63 (8.85, 10.41)	3.60 (3.17, 4.02)	13.61 (12.08, 15.15)	4.78 (4.3, 5.26)	8.17 (7.15, 9.19)	3.37 (3.18, 3.56)	219.36 (186.5, 252.23)	75.65 (66.51, 84.79)
	2028	9.63 (8.75, 10.51)	3.63 (3.08, 4.17)	13.53 (11.61, 15.44)	4.77 (4.16, 5.37)	8.17 (7.03, 9.31)	3.37 (3.18, 3.56)	219.43 (183.16, 255.71)	75.65 (65.40, 85.89)
	2029	9.63 (8.69, 10.57)	3.66 (2.98, 4.33)	13.45 (11.14, 15.75)	4.75 (4.01, 5.50)	8.17 (6.92, 9.42)	3.37 (3.18, 3.56)	219.44 (180.19, 258.68)	75.64 (64.39, 86.89)
	2030	9.63 (8.65, 10.61)	3.69 (2.87, 4.50)	13.37 (10.66, 16.08)	4.74 (3.84, 5.64)	8.17 (6.82, 9.52)	3.37 (3.18, 3.56)	219.41 (177.48, 261.34)	75.64 (63.46, 87.82)
	2031	9.64 (8.64, 10.64)	3.72 (2.75, 4.68)	13.29 (10.16, 16.43)	4.73 (3.67, 5.79)	8.17 (6.73, 9.61)	3.37 (3.18, 3.56)	219.38 (174.96, 263.8)	75.64 (62.59, 88.69)
	2032	9.65 (8.65, 10.66)	3.75 (2.62, 4.88)	13.22 (9.63, 16.81)	4.72 (3.49, 5.94)	8.17 (6.64, 9.70)	3.37 (3.18, 3.56)	219.36 (172.58, 266.14)	75.64 (61.77, 89.51)
	2033	9.67 (8.66, 10.68)	3.78 (2.49, 5.07)	13.15 (9.08, 17.21)	4.70 (3.30, 6.11)	8.17 (6.56, 9.78)	3.37 (3.18, 3.55)	219.35 (170.31, 268.39)	75.64 (61.00, 90.28)
	2034	9.68 (8.67, 10.69)	3.81 (2.34, 5.28)	13.07 (8.51, 17.63)	4.69 (3.10, 6.28)	8.17 (6.48, 9.86)	3.36 (3.17, 3.55)	219.35 (168.14, 270.56)	75.64 (60.27, 91.01)
	2035	9.68 (8.67, 10.69)	3.84 (2.19, 5.50)	13.00 (7.92, 18.07)	4.68 (2.90, 6.46)	8.17 (6.40, 9.93)	3.36 (3.17, 3.55)	219.35 (166.06, 272.64)	75.64 (59.57, 91.71)
	2036	9.68 (8.67, 10.69)	3.87 (2.03, 5.72)	12.92 (7.31, 18.52)	4.67 (2.69, 6.65)	8.17 (6.33, 10.00)	3.36 (3.17, 3.55)	219.35 (164.05, 274.66)	75.64 (58.90, 92.38)

G20, group of 20; CI, confidence interval; DALYs, disability-adjusted life years; ASIR, the age-standardized incidence rate; ASPR, the age-standardized prevalence rate; ASMR, age-standardized mortality rate; ASDR, age-standardized DALYs rate.

## Discussion

Liver cancer represents a severe global threat to human health and constitutes a major public health challenge. China carries one of the world's highest burdens of this disease (1, 20). The GBD study provides essential data for examining disease burden, forecasting trends, and enabling cross-national comparisons (21). A comprehensive evaluation of the long-term development trajectory of liver cancer in China, alongside comparative analysis with other countries, is crucial for informing domestic public health policy formulation, strengthening international collaboration on disease prevention and control, and enhancing population health outcomes. However, rigorous comparative analyses of liver cancer burden between China and other countries remain scarce, and the most recent trends alongside future projections for China are still not thoroughly examined. Leveraging the latest GBD 2021 database, this study pioneers a comprehensive assessment of liver cancer incidence, prevalence, mortality, and DALYs trends from 1990 to 2021 in China and G20 countries (representing the world's largest economies), stratified by age group and sex. Furthermore, projections for the period 2022–2036 are provided. This analysis serves as a critical reference for optimizing liver cancer prevention and control strategies globally.

Our analysis indicates declining trends in China's ASIR, ASPR, ASMR, and ASDR for liver cancer from 1990 to 2021, with particularly significant reductions in ASMR and ASDR. In contrast, G20 countries exhibited heterogeneous patterns: ASIR and ASPR increased, ASMR decreased marginally, while ASDR declined substantially. Notably, the China-to-G20 ASDR ratio decreased from 1.95 (334.52 vs. 171.28 per 100,000 people) in 1990 to 1.64 (239.91 vs. 146.24 per 100,000 people) in 2021, reflecting a narrowing gap in liver cancer burden.

We speculate that this reduction may be due to three factors. First, China's rapid economic growth over the past decades has driven socioeconomic and healthcare advancements. These advancements have elevated living standards and health awareness (22, 23), thereby reducing ASIR, ASPR, ASMR, and ASDR. Second, public health strategies, particularly HBV vaccination, drove sharp declines of ASIR, ASPR, ASMR, and ASDR during 2000–2005. HBV vaccine coverage exceeded 90% after its 1992 inclusion in the national immunization program and full subsidy implementation in 2002 (24), curtailing HBV transmission and hepatocellular carcinoma risk. Recent integration of antiviral therapies (e.g., entecavir, tenofovir) into China Healthcare Security further reduced cirrhosis and cancer incidence among chronic HBV patients. Third, long-standing public health policies are likely contributors to the observed trends. For example, the tobacco control measures initiated in 2005 have potentially contributed to a reduced incidence of tobacco-related cancers over the long term (25). More recently, initiatives such as the 2025 Healthy Weight Management Initiative represent a continued focus on mitigating obesity-related carcinogenesis, though their impact on population-level cancer mortality rates would be expected to manifest in the future beyond the current study period (26). Enhanced food safety regulations, industrial emission controls, and environmental policies also reduced carcinogen exposure (27, 28). These findings align with previous research demonstrating significant reductions in Asia's ASPR, particularly in countries like China where successful implementation of HBV immunization programs and complementary public health interventions occurred (29–31).

Despite three decades of declining incidence, prevalence, mortality, and DALYs, China's liver cancer burden remains substantial due to its large population and aging demographics (32, 33). Critically, China's ASIR, ASPR, ASMR, and ASDR in 1990 and 2021 significantly exceeded the average of G20 countries. The persistently higher burden in China may be driven by several underlying factors: Firstly, China was once a high-prevalence area for HBV, with a large historical population base of infected individuals, and HBV infection and aflatoxin exposure (related to dietary habits and grain storage conditions) are thought to have a synergistic carcinogenic effect. Secondly, unique dietary cultures (such as high sodium intake and low fruit consumption) and drinking customs further amplify the risk of chronic liver diseases. Thirdly, environmental pollution during rapid industrialization and urbanization (such as PM2.5 pollution) continues to exacerbate the disease burden. Fourthly, although the healthcare system has been continuously improving, there remains room for enhancement in chronic disease prevention and control (especially in primary screening coverage and standardization), public health awareness promotion, and multi-department collaborative policy guidance, leading to some preventable risks remaining uncontrolled. Furthermore, the observed trends in G20 countries may be partly attributable to advancements in screening and early detection protocols adopted by several member states. For instance, the American Association for the Study of Liver Diseases (AASLD) 2023 guidelines, which recommend screening high-risk metabolic dysfunction-associated steatotic liver disease (MASLD) patients using GALAD scores, represent a targeted approach to identifying pre-neoplastic conditions. Similarly, Italy's broadened HCV screening initiatives aim to reduce the burden of virus-related hepatocarcinogenesis by improving early diagnosis (34). While implementation varies across the G20 countries, the adoption of such evidence-based, precision screening strategies by key economies may collectively contribute to the earlier stage diagnosis and improved survival rates reflected in the overall data for the group. While our data show a decline in liver cancer burden, the relative contributions of specific factors such as HBV vaccination, healthcare improvements, and economic development remain to be fully elucidated through future studies employing causal decomposition models or individual-level policy-exposure data.

The burden of liver cancer also showed gender and age-based differences. Liver cancer risk escalates significantly from ages 35–85, peaking at 80–84 years, likely due to prolonged exposure to chronic liver disease, HBV/HCV infection, alcohol, and smoking. Declining incidence after age 85 may reflect competing mortality risks (35–37). Male ASIR, ASPR, ASMR, and ASDR consistently exceeded female rates in both China and G20 countries (1990 and 2021), attributable to genetic, environmental, and behavioral factors (e.g., smoking/alcohol use) (38). Estrogen's protective effects (39, 40) and androgen-related mechanisms [e.g., enhanced dehydroepiandrosterone (DHEA) conversion] (41) may also contribute to this disparity. However, females over 85 showed higher case numbers and mortality, potentially due to longer lifespans, cumulative liver disease exposure, and inadequate geriatric care (28, 42–44).

Joinpoint regression confirmed overall declines in China's ASIR, ASDR, and ASMR during 1990–2021 but detected an uptrend from 1995–2000, potentially linked to expanded screening or cancer registry development (45). The National Cancer Center Registry of China was established as the National Cancer Registry Administration in 2002, which may have led to a short-term increase in the number of reported cases.

ARIMA modeling predicts rising ASIR for both sexes in China and G20 countries (2022–2036), while ASDR will remain stable except for a decline among Chinese females. These trends suggest that liver cancer burdens in both China and G20 countries will persist at elevated levels without substantial near-term mitigation. Consequently, enhancing evidence-based strategies for prevention and early detection is imperative, particularly prioritizing high-risk population screening—such as young adults with liver cancer diagnoses among first-degree relatives. Our forecasts are subject to uncertainty arising from post-2021 events. Disruptions to healthcare systems during the COVID-19 pandemic may have delayed diagnoses (46, 47), while the expanded use of direct-acting antivirals could accelerate the decline in HCV-related liver cancer faster than projected. Conversely, the rapidly growing epidemic of MASLD (48) may lead to an increase in cases beyond what our model, based on pre-2021 data, predicts. These factors highlight the need for future updates to our forecasts to incorporate more recent data. Therefore, subsequent forecasts should incorporate real-time data on these evolving factors to enhance their precision and public health relevance.

This study utilized the latest GBD 2021 data to comparatively analyze the age- and sex-stratified burden of liver cancer in China and G20 countries, projecting trends through 2036 to inform prevention strategies. However, several limitations in this study warrant acknowledgment. Firstly, regarding methodological constraints, the reliability of GBD 2021 estimates may be compromised in certain regions as they rely on modeling derived from public health reports and mortality registries. This is particularly relevant in low- and middle-income countries where incomplete data could affect the accuracy of burden assessments. Secondly, pertaining to our modeling approach, the ARIMA model primarily relies on historical time trends and does not incorporate potential future changes in policy interventions, emerging risk factors, or public health initiatives. This limitation may reduce the model's accuracy in predicting long-term disease burden trajectories, as it cannot account for non-linear disruptions or novel epidemiological shifts. Thirdly, we acknowledge the geopolitical and socioeconomic considerations: while the G20 provides a useful framework for analysis, it represents a highly heterogeneous group of nations with substantial differences in healthcare systems, economic development, and epidemiological profiles. Our aggregated “group-level” analyses may have masked important between-country differences, potentially obscuring distinctive patterns that might be relevant for targeted public health interventions. Fourthly, we recognize the constraint of statistical timeliness; the current GBD database only extends to 2021, resulting in a lack of up-to-date disease burden statistics for recent years. Finally, we note an important mechanistic pathway limitation: the interplay between genetic predispositions and environmental risk factors in liver cancer pathogenesis was unexplored in our analysis, leaving critical mechanistic pathways unexamined that might help explain observed epidemiological patterns.

## Conclusion

Collectively, this study provides comprehensive longitudinal analysis and projections of liver cancer burden in China and G20 countries from 1990 through 2036. During 1990–2021, China exhibited significant reductions in ASIR, ASPR, ASMR, and ASDR, consistent with implementation of preventive measures, healthcare advancements, and improved economic conditions. Notwithstanding these gains, China maintains substantially higher rates compared to contemporaneous G20 levels. Projections indicate limited mitigation of China's liver cancer burden over the next 15 years. Although considerable progress has been achieved in alleviating this malignancy's impact across both China and G20 countries, sustained efforts remain essential to address persisting challenges—particularly in risk factor modulation targeting high-risk subgroups and equitable healthcare access. Global initiatives should consequently prioritize evidence-based prevention initiatives for populations and regions exhibiting escalating burden trajectories. Future studies should pursue three critical pathways: monitoring evolving epidemiological trends, refining early-detection and therapeutic approaches, and developing novel biomarker-driven prevention strategies alongside precision technologies for liver cancer to achieve substantive progress in disease control.

## Data Availability

The original contributions presented in the study are included in the article/Supplementary material, further inquiries can be directed to the corresponding authors.

## References

[1] Bray F Laversanne M Sung H Ferlay J Siegel RL Soerjomataram I . Global cancer statistics 2022: GLOBOCAN estimates of incidence and mortality worldwide for 36 cancers in 185 countries. CA Cancer J Clin. (2024) 74:229–63. doi: 10.3322/caac.21834 38572751

[2] McGlynn KA Petrick JL London WT. Global epidemiology of hepatocellular carcinoma: an emphasis on demographic and regional variability. Clin Liver Dis. (2015) 19:223–38. doi: 10.1016/j.cld.2015.01.001 25921660 PMC4712629

[3] Shi JF Cao M Wang Y Bai FZ Lei L Peng J . Is it possible to halve the incidence of liver cancer in China by 2050? Int J Cancer. (2021) 148:1051–65. doi: 10.1002/ijc.33313 32997794

[4] Collaborators GD. Global age-sex-specific fertility, mortality, healthy life expectancy (HALE), and population estimates in 204 countries and territories, 1950–2019: a comprehensive demographic analysis for the Global Burden of Disease Study 2019. Lancet. (2020) 396:1160–203. doi: 10.1016/S0140-6736(20)30977-6 33069325 PMC7566045

[5] GBD 2019 Diseases and Injuries Collaborators. Global burden of 369 diseases and injuries in 204 countries and territories, 1990–2019: a systematic analysis for the Global Burden of Disease Study 2019. Lancet. (2020) 396:1204–22. doi: 10.1016/S0140-6736(20)30925-9 33069326 PMC7567026

[6] Liu Y Zheng J Hao J Wang RR Liu X Gu P . Global burden of primary liver cancer by five etiologies and global prediction by 2035 based on global burden of disease study 2019. Cancer Med. (2022) 11:1310–23. doi: 10.1002/cam4.4551 35118819 PMC8894698

[7] Valery PC Laversanne M Clark PJ Petrick JL McGlynn KA Bray F. Projections of primary liver cancer to 2030 in 30 countries worldwide. Hepatology. (2018) 67:600–11. doi: 10.1002/hep.29498 28859220 PMC5832532

[8] Dieleman JL Cowling K Agyepong IA Alkenbrack S Bollyky TJ Bump JB . The G20 and development assistance for health: historical trends and crucial questions to inform a new era. Lancet. (2019) 394:173–83. doi: 10.1016/S0140-6736(19)31333-9 31257126

[9] McBride B Hawkes S Buse K. Soft power and global health: the sustainable development goals (SDGs) era health agendas of the G7, G20 and BRICS. BMC Public Health. (2019) 19:815. doi: 10.1186/s12889-019-7114-5 31234831 PMC6591917

[10] Xing QQ Li JM Dong X Zeng DY Chen ZJ Lin XY . Socioeconomics and attributable etiology of primary liver cancer, 1990–2019. World J Gastroenterol. (2022) 28:2361–82. doi: 10.3748/wjg.v28.i21.2361 35800181 PMC9185214

[11] Gnyawali B Pusateri A Nickerson A Jalil S Mumtaz K. Epidemiologic and socioeconomic factors impacting hepatitis B virus and related hepatocellular carcinoma. World J Gastroenterol. (2022) 28:3793–802. doi: 10.3748/wjg.v28.i29.3793 36157533 PMC9367226

[12] Liu C Zhu S Zhang J Wu P Wang X Du S . Global, regional, and national burden of liver cancer due to non-alcoholic steatohepatitis, 1990–2019: a decomposition and age-period-cohort analysis. J Gastroenterol. (2023) 58:1222–36. doi: 10.1007/s00535-023-02040-4 37665532

[13] Akinyemiju T Abera S Ahmed M Alam N Alemayohu MA Allen C . The burden of primary liver cancer and underlying etiologies from 1990 to 2015 at the global, regional, and national level: results from the global burden of disease STUDY 2015. JAMA Oncol. (2017) 3:1683–91. doi: 10.1001/jamaoncol.2017.3055 28983565 PMC5824275

[14] Long J Cui K Wang D Qin S Li Z. Burden of hepatocellular carcinoma and its underlying etiologies in China, 1990–2021: findings from the global burden of disease study 2021. Cancer Control. (2024) 31:10732748241310573. doi: 10.1177/10732748241310573 39703050 PMC11660275

[15] Yang S Deng Y Zheng Y Zhang J He D Dai Z . Burden, trends, and predictions of liver cancer in China, Japan, and South Korea: analysis based on the Global Burden of Disease Study 2021. Hepatol Int. (2025) 19:441–59. doi: 10.1007/s12072-024-10763-6 39799268 PMC12003535

[16] Kim HJ Fay MP Feuer EJ Midthune DN. Permutation tests for joinpoint regression with applications to cancer rates. Stat Med. (2000) 19:335–51. doi: 10.1002/(sici)1097-0258(20000215)19:3<335::aid-sim336>3.0.co;2-z 10649300

[17] Qiu H Cao S Xu R. Cancer incidence, mortality, and burden in China: a time-trend analysis and comparison with the United States and United Kingdom based on the global epidemiological data released in 2020. Cancer Commun. (2021) 41:1037–48. doi: 10.1002/cac2.12197 34288593 PMC8504144

[18] Shi L Bao C Wen Y Liu X You G. Analysis and comparison of the trends in burden of rheumatic heart disease in China and worldwide from 1990 to 2019. BMC Cardiovasc Disord. (2023) 23:517. doi: 10.1186/s12872-023-03552-w 37875798 PMC10594932

[19] Li Y Ning Y Shen B Shi Y Song N Fang Y . Temporal trends in prevalence and mortality for chronic kidney disease in China from 1990 to 2019: an analysis of the Global Burden of Disease Study 2019. Clin Kidney J. (2023) 16:312–21. doi: 10.1093/ckj/sfac218 36755850 PMC9900593

[20] Han B Zheng R Zeng H Wang S Sun K Chen R . Cancer incidence and mortality in China, 2022. J Natl Cancer Cent. (2024) 4:47–53. doi: 10.1016/j.jncc.2024.01.006 39036382 PMC11256708

[21] GBD 2019 Risk Factors Collaborators. Global burden of 87 risk factors in 204 countries and territories, 1990–2019: a systematic analysis for the Global Burden of Disease Study 2019. Lancet. (2020) 396:1223–49. doi: 10.1016/S0140-6736(20)30752-2 33069327 PMC7566194

[22] Wang SM Zheng RS Zhang SW Zeng HM Chen R Sun KX . [Epidemiological characteristics of gastric cancer in China, 2015]. Zhonghua Liu Xing Bing Xue Za Zhi. (2019) 40:1517–21. doi: 10.3760/cma.j.issn.0254-6450.2019.12.003 32062908

[23] Yang P Huang W Xu Y Teng Y Shu P. Trends and projections of the burden of gastric cancer in China and G20 countries: a comparative study based on the global burden of disease database 2021. Int J Surg. (2025) 111:4854–65. doi: 10.1097/JS9.0000000000002464 40359560

[24] Wang Z Chen Y Pan J. Trends of acute hepatitis B notification rates in eastern China from 2005 to 2013. PLoS ONE. (2014) 9:e114645. doi: 10.1371/journal.pone.0114645 25504088 PMC4264791

[25] Yang G Wang Y Wu Y Yang J Wan X. The road to effective tobacco control in China. Lancet. (2015) 385:1019–28. doi: 10.1016/S0140-6736(15)60174-X 25784349

[26] Sun X Sun Z Peng W Zhang J Gou B Tian X . Expert consensus and call on actions for weight management in China: advancing healthy china initiative through strategic actions. China CDC Weekly. (2024) 6:1347–53. doi: 10.46234/ccdcw2024.268 39802088 PMC11724133

[27] Fitzmaurice C Abate D Abbasi N Abbastabar H Abd-Allah F Abdel-Rahman O . Global, regional, and national cancer incidence, mortality, years of life lost, years lived with disability, and disability-adjusted life-years for 29 cancer groups, 1990 to 2017: a systematic analysis for the global burden of disease study. JAMA Oncol. (2019) 5:1749–68. doi: 10.1001/jamaoncol.2019.2996 31560378 PMC6777271

[28] Yang JD Hainaut P Gores GJ Amadou A Plymoth A Roberts LR . Global view of hepatocellular carcinoma: trends, risk, prevention and management. Nat Rev Gastroenterol Hepatol. (2019) 16:589–604. doi: 10.1038/s41575-019-0186-y 31439937 PMC6813818

[29] Yang C Jia J Yu Y Lu H Zhang L. Temporal trends in prevalence of liver cancer and etiology-specific liver cancer from 1990 to 2019. Clin Res Hepatol Gastroenterol. (2024) 48:102451. doi: 10.1016/j.clinre.2024.102451 39174005

[30] Sung H Ferlay J Siegel RL Laversanne M Soerjomataram I Jemal A . Global cancer statistics 2020: GLOBOCAN estimates of incidence and mortality worldwide for 36 cancers in 185 countries. CA Cancer J Clin. (2021) 71:209–49. doi: 10.3322/caac.21660 33538338

[31] Baecker A Liu X La Vecchia C Zhang ZF. Worldwide incidence of hepatocellular carcinoma cases attributable to major risk factors. Eur J Cancer Prev. (2018) 27:205–12. doi: 10.1097/CEJ.0000000000000428 29489473 PMC5876122

[32] Cao G Liu J Liu M. Global, regional, and national trends in incidence and mortality of primary liver cancer and its underlying etiologies from 1990 to 2019: results from the global burden of disease study 2019. J Epidemiol Glob Health. (2023) 13:344–60. doi: 10.1007/s44197-023-00109-0 37178451 PMC10271958

[33] Liu Z Mao X Jiang Y Cai N Jin L Zhang T . Changing trends in the disease burden of primary liver cancer caused by specific etiologies in China. Cancer Med. (2019) 8:5787–99. doi: 10.1002/cam4.2477 31385465 PMC6745850

[34] Kondili LA Craxì L Nava F Babudieri S D'Ambrosio R Marcellusi A . From prioritization to universal treatment: successes and challenges of hepatitis C virus elimination in Italy. J Infect Dis. (2023) 228:S211–20. doi: 10.1093/infdis/jiad038 37703346

[35] Torre LA Bray F Siegel RL Ferlay J Lortet-Tieulent J Jemal A. Global cancer statistics, 2012. CA Cancer J Clin. (2015) 65:87–108. doi: 10.3322/caac.21262 25651787

[36] Chidambaranathan-Reghupaty S Fisher PB Sarkar D. Hepatocellular carcinoma (HCC): epidemiology, etiology and molecular classification. Adv Cancer Res. (2021) 149:1–61. doi: 10.1016/bs.acr.2020.10.001 33579421 PMC8796122

[37] El-Serag HB. Epidemiology of viral hepatitis and hepatocellular carcinoma. Gastroenterology. (2012) 142:1264–73.e1. doi: 10.1053/j.gastro.2011.12.061 22537432 PMC3338949

[38] Siegel RL Miller KD Wagle NS Jemal A. Cancer statistics, 2023. CA Cancer J Clin. (2023) 73:17–48. doi: 10.3322/caac.21763 36633525

[39] Iavarone M Lampertico P Seletti C Francesca Donato M Ronchi G. del Ninno E, et al. The clinical and pathogenetic significance of estrogen receptor-beta expression in chronic liver diseases and liver carcinoma. Cancer. (2003) 98:529–34. doi: 10.1002/cncr.11528 12879470

[40] Tan DJH Setiawan VW Ng CH Lim WH Muthiah MD Tan EX . Global burden of liver cancer in males and females: changing etiological basis and the growing contribution of NASH. Hepatology. (2023) 77:1150–63. doi: 10.1002/hep.32758 36037274

[41] Wu Z Petrick JL Florio AA Guillemette C Beane Freeman LE Buring JE . Endogenous sex steroid hormones and risk of liver cancer among US men: results from the liver cancer pooling project. JHEP Rep. (2023) 5:100742. doi: 10.1016/j.jhepr.2023.100742 37425211 PMC10326694

[42] Al Ta'ani O Al-Ajlouni Y Jagdish B Khataniar H Aleyadeh W Al-Bitar F . Examining the evolving landscape of liver cancer burden in the United States from 1990 to 2019. BMC Cancer. (2024) 24:1098. doi: 10.1186/s12885-024-12869-4 39232707 PMC11373298

[43] Kulik L El-Serag HB. Epidemiology and management of hepatocellular carcinoma. Gastroenterology. (2019) 156:477–91.e1. doi: 10.1053/j.gastro.2018.08.065 30367835 PMC6340716

[44] Couri T Pillai A. Goals and targets for personalized therapy for HCC. Hepatol Int. (2019) 13:125–37. doi: 10.1007/s12072-018-9919-1 30600478

[45] Wei W Zeng H Zheng R Zhang S An L Chen R . Cancer registration in China and its role in cancer prevention and control. Lancet Oncol. (2020) 21:e342–9. doi: 10.1016/S1470-2045(20)30073-5 32615118

[46] Chen C Zhou W Cui Y Cao K Chen M Qu R . Global, regional, and national characteristics of the main causes of increased disease burden due to the covid-19 pandemic: time-series modelling analysis of global burden of disease study 2021. BMJ. (2025) 390:e083868. doi: 10.1136/bmj-2024-083868 40602809 PMC12216812

[47] Global age-sex-specific mortality life expectancy population estimates in 204 countries territories 811 subnational locations 1950-2021 and and the impact of the COVID-19 pandemic: a comprehensive demographic analysis for the Global Burden of Disease Study 2021. Lancet. (2024) 403:1989–2056. doi: 10.1016/S0140-6736(24)00476-8 38484753 PMC11126395

[48] Koo TH Chowdhary R Lee YL Leong XB Zakaria AD. What is the difference between metabolic dysfunction-associated steatotic liver disease, eosinophilic esophagitis and gastroesophageal reflux disease? Med J Malaysia. (2025) 80:533–6. Available online at: https://www.e-mjm.org/2025/v80n5/MASLD-EoE-GERD.pdf 41015992

