# Supplementary material for: Burden, trends and projections of liver cancer in China and G20 countries: a comparative study based on the global burden of disease database 2021
Source: Front Public Health. 2026 Jan 16;13:1668750. doi: 10.3389/fpubh.2025.1668750 (PMC12857313; doi:10.3389/fpubh.2025.1668750)
Supplement: Supplementary file 1 [file Data_Sheet_1.zip › Supplementary/Supplementary 3.docx]

**Supplementary 3 The APC of age-standardized incidence, prevalence,**

**mortality, DALYs, and corresponding AAPC of Liver Cancer in China and**

**G20 Countries in 1990 and 2021**

| **Location** | **Measure**  **(per 100,000 people)** | **APC** | | | | | **1990-2021 AAPC** |
| --- | --- | --- | --- | --- | --- | --- | --- |
|  |  | **Segment** | | **N (95% CI)** | | ***P*.Value** | **N (95% CI)** |
| **China** | **ASIR** | 1990-1995 | | -0.19 (-0.43 - 0.05) | | 0.115509 | -0.31  (-0.39 - -0.23) |
|  |  | 1995-2000 | | 1.60 (1.35 - 1.86) | | 0 |  |
|  |  | 2000-2005 | | -3.39 (-3.60 - -3.19) | | 0 |  |
|  |  | 2005-2016 | | 0.83 (0.78 - 0.89) | | 0 |  |
|  |  | 2016-2021 | | -1.70 (-1.99 - -1.40) | | 0 |  |
|  | **ASPR** | 1990-1995 | | 0.55 (0.17 - 0.93) | | 0.007587 | 0.02  (-0.11 - 0.15) |
|  |  | 1995-2000 | | 1.74 (1.33 - 2.16) | | 0 |  |
|  |  | 2000-2005 | | -3.94 (-4.26 - -3.62) | | 0 |  |
|  |  | 2005-2010 | | 0.87 (0.56 - 1.18) | | 2.20E-05 |  |
|  |  | 2010-2015 | | 1.81 (1.42 - 2.20) | | 0 |  |
|  |  | 2015-2021 | | -0.65 (-0.98 - -0.33) | | 0.000705 |  |
|  | **ASMR** | 1990-2001 | | 0.53 (0.24 - 0.82) | | 0.001403 | -0.68  (-1.25 - -0.10) |
|  |  | 2001-2006 | | -3.77 (-4.69 - -2.83) | | 0 |  |
|  |  | 2006-2009 | | 1.50 (-1.78 - 4.89) | | 0.348766 |  |
|  |  | 2009-2012 | | -1.75 (-4.87 - 1.48) | | 0.262435 |  |
|  |  | 2012-2021 | | 2.12 (-1.60 - 5.97) | | 0.247137 |  |
|  | **ASDR** | 1990-2001 | | 0.39 (0.18 - 0.59) | | 0.001137 | -0.96  (-1.36 –  -0.56) |
|  |  | 2001-2005 | | -5.32 (-6.36 - -4.27) | | 0 |  |
|  |  | 2005-2009 | | -0.13 (-1.19 - 0.94) | | 0.797492 |  |
|  |  | 2009-2012 | | -2.06 (-4.41 - 0.35) | | 0.087861 |  |
|  |  | 2012-2015 | | 2.06 (-0.76 - 4.95) | | 0.14179 |  |
|  |  | 2015-2021 | | -1.92 (-2.63 - -1.20) | | 4.40E-05 |  |
| **G20** | **ASIR** | 1990-2000 | | 1.56 (1.51 - 1.61) | | 0 | 0.20  (0.15 - 0.24) |
|  |  | 2000-2005 | | -1.54 (-1.70 - -1.37) | | 0 |  |
|  |  | 2005-2015 | | 0.52 (0.47 - 0.57) | | 0 |  |
|  |  | 2015-2021 | | -1.12 (-1.28 - -0.96) | | 0 |  |
|  | **ASPR** | 1990-2000 | | 2.09 (2.05 - 2.14) | | 0 | 0.50  (0.46 - 0.54) |
|  |  | 2000-2005 | | -0.87 (-0.99 - -0.74) | | 0 |  |
|  |  | 2005-2009 | | 0.70 (0.51 - 0.88) | | 0 |  |
|  |  | 2009-2015 | | 0.38 (0.28 - 0.48) | | 0 |  |
|  |  | 2015-2021 | | -0.99 (-1.10 - -0.87) | | 0 |  |
|  | **ASMR** | 1990-2000 | | 1.42 (1.20 - 1.64) | | 0 | -0.04  (-0.24 - 0.15) |
|  |  | 2000-2005 | | -1.99 (-2.67 - -1.30) | | 6.00E-06 |  |
|  |  | 2005-2016 | | 0.05 (-0.14 - 0.24) | | 0.582389 |  |
|  |  | 2016-2021 | | -1.19 (-2.07 - -0.30) | | 0.011463 |  |
|  | **ASDR** | 1990-2000 | 1.05 (0.88 - 1.21) | | 0 | | -0.46  (-0.72 - -0.19) |
|  |  | 2000-2005 | -2.87 (-3.34 - -2.39) | | 0 | |  |
|  |  | 2005-2009 | -0.11 (-0.84 - 0.64) | | 0.761983 | |  |
|  |  | 2009-2012 | -1.27 (-2.85 - 0.34) | | 0.113343 | |  |
|  |  | 2012-2015 | 0.87 (-1.00 - 2.76) | | 0.339863 | |  |
|  |  | 2015-2021 | -1.40 (-1.88 - -0.92) | | 1.70E-05 | |  |

G20, Group of 20; CI, confidence interval; DALYs, disability-adjusted life years; APC, annual percent change; AAPC, average annual percentage change
