# Supplementary material for: Burden, trends and projections of liver cancer in China and G20 countries: a comparative study based on the global burden of disease database 2021
Source: Front Public Health. 2026 Jan 16;13:1668750. doi: 10.3389/fpubh.2025.1668750 (PMC12857313; doi:10.3389/fpubh.2025.1668750)
Supplement: Supplementary file 1 [file Data_Sheet_1.zip › Supplementary/Supplementary 4/Table .docx]

**Table Summary of Sex × Year Interaction Results for Mortality Indicators in China and G20 Countries**

| **Indication** | **Location** | **β (Coefficient)** | **P-value (Interaction)** | **Female Annual Change (/year)** | **Male Annual Change (/year)** | **R²** | **Adjusted R²** | **F-statistic (df=3,58)** | **Model**  **P-value** |
| --- | --- | --- | --- | --- | --- | --- | --- | --- | --- |
| ASIR | China | 0.01 | 0.37 | -0.03 | -0.02 | 0.99 | 0.99 | 2,513.60 | <0.001 |
| ASPR |  | 0.07 | 0.00 | -0.04 | +0.03 | 0.99 | 0.99 | 1,551.00 | <0.001 |
| ASMR |  | -0.05 | 0.00 | -0.05 | -0.09 | 0.99 | 0.99 | 1,807.00 | <0.001 |
| ASDR |  | -2.24 | 0.00 | -2.20 | -4.44 | 0.99 | 0.99 | 1,728.10 | <0.001 |
| ASIR | G20 | 0.00 | 0.47 | +0.00 | +0.01 | 0.99 | 0.99 | 3,630.40 | <0.001 |
| ASPR |  | 0.06 | 0.00 | +0.01 | +0.07 | 0.99 | 0.99 | 2,692.50 | <0.001 |
| ASMR |  | -0.02 | 0.01 | -0.00 | -0.02 | 0.99 | 0.99 | 2,545.80 | <0.001 |
| ASDR |  | -1.17 | 0.00 | -0.53 | -1.70 | 0.99 | 0.99 | 2,655.90 | <0.001 |

β denotes the sex × year interaction coefficient, with significant *P*-values (*P* < 0.05) indicating diverging trends by gender; annual changes are gender-specific mortality rate slopes; model diagnostics include R² and adjusted R² for goodness-of-fit, and F-statistics for overall model significance. G20, Group of 20; ASIR, the age-standardized incidence rate; ASPR, the age-standardized prevalence rate; ASMR, age-standardized mortality rate; ASDR, age-standardized DALYs rate
