# Supplementary figures and images for: Burden, trends and projections of liver cancer in China and G20 countries: a comparative study based on the global burden of disease database 2021
Source: Front Public Health. 2026 Jan 16;13:1668750. doi: 10.3389/fpubh.2025.1668750 (PMC12857313; doi:10.3389/fpubh.2025.1668750)

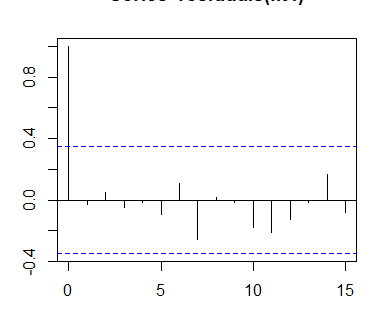

Supplement: Supplementary file 1 [file Data_Sheet_1.zip › Supplementary/Supplementary 2/ARIMA-ACF/China/female-DALY.tiff]

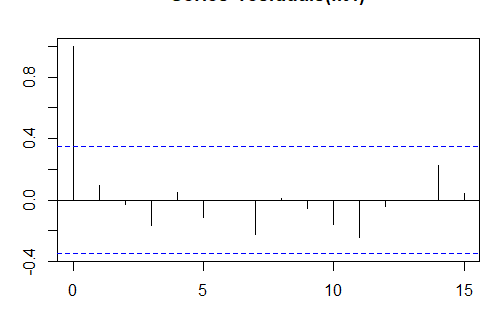

Supplement: Supplementary file 1 [file Data_Sheet_1.zip › Supplementary/Supplementary 2/ARIMA-ACF/China/female-de.tiff]

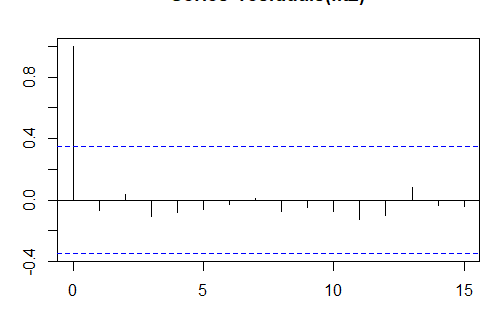

Supplement: Supplementary file 1 [file Data_Sheet_1.zip › Supplementary/Supplementary 2/ARIMA-ACF/China/female-in.tiff]

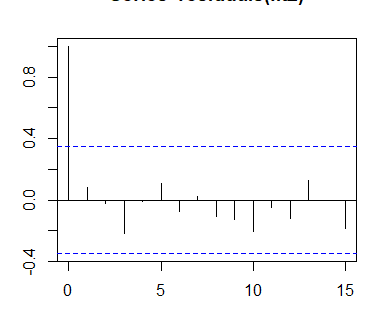

Supplement: Supplementary file 1 [file Data_Sheet_1.zip › Supplementary/Supplementary 2/ARIMA-ACF/China/female-pr.tiff]

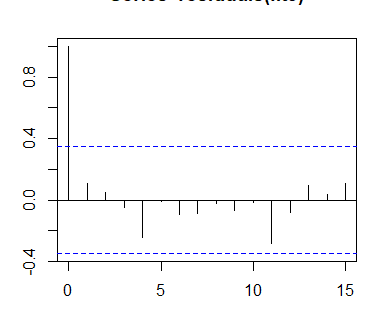

Supplement: Supplementary file 1 [file Data_Sheet_1.zip › Supplementary/Supplementary 2/ARIMA-ACF/China/male-DALY.tiff]

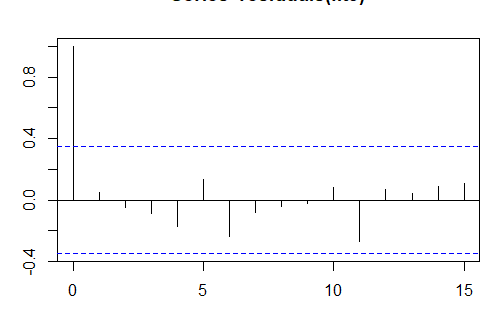

Supplement: Supplementary file 1 [file Data_Sheet_1.zip › Supplementary/Supplementary 2/ARIMA-ACF/China/male-de.tiff]

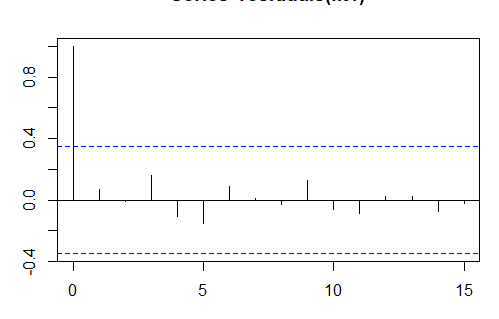

Supplement: Supplementary file 1 [file Data_Sheet_1.zip › Supplementary/Supplementary 2/ARIMA-ACF/China/male-in.tiff]

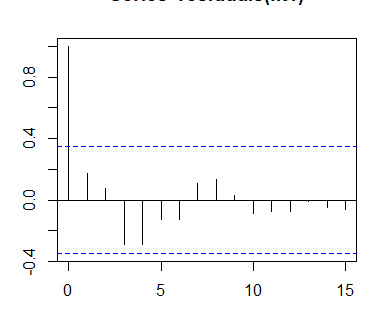

Supplement: Supplementary file 1 [file Data_Sheet_1.zip › Supplementary/Supplementary 2/ARIMA-ACF/China/male-pr.tiff]

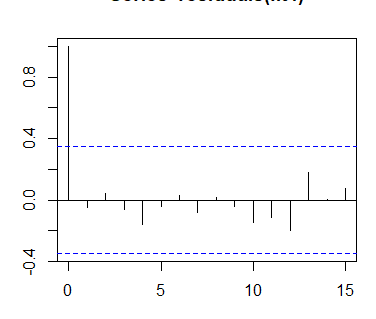

Supplement: Supplementary file 1 [file Data_Sheet_1.zip › Supplementary/Supplementary 2/ARIMA-ACF/G20/female-DALY.tiff]

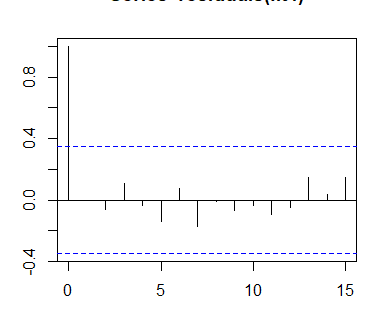

Supplement: Supplementary file 1 [file Data_Sheet_1.zip › Supplementary/Supplementary 2/ARIMA-ACF/G20/female-de.tiff]

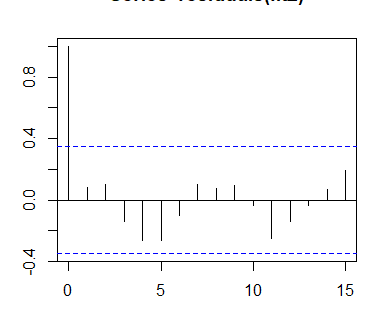

Supplement: Supplementary file 1 [file Data_Sheet_1.zip › Supplementary/Supplementary 2/ARIMA-ACF/G20/female-in.tiff]

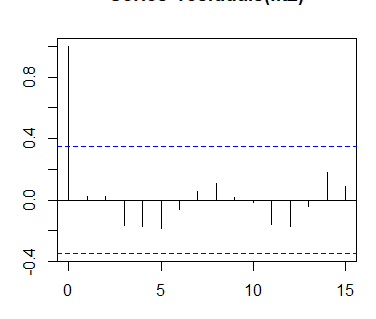

Supplement: Supplementary file 1 [file Data_Sheet_1.zip › Supplementary/Supplementary 2/ARIMA-ACF/G20/female-pr.tiff]

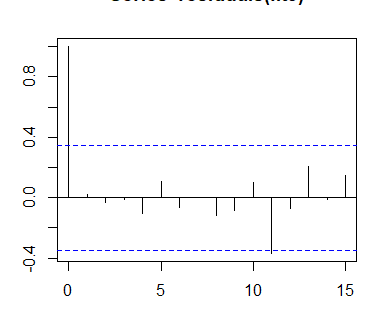

Supplement: Supplementary file 1 [file Data_Sheet_1.zip › Supplementary/Supplementary 2/ARIMA-ACF/G20/male-DALY.tiff]

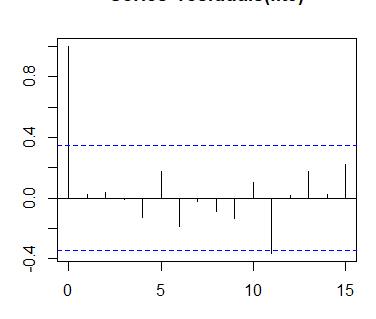

Supplement: Supplementary file 1 [file Data_Sheet_1.zip › Supplementary/Supplementary 2/ARIMA-ACF/G20/male-de.tiff]

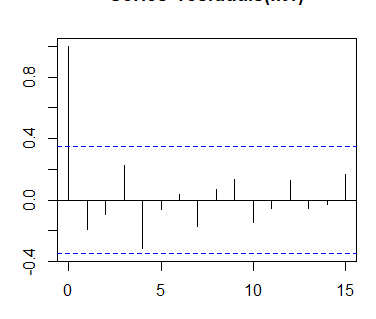

Supplement: Supplementary file 1 [file Data_Sheet_1.zip › Supplementary/Supplementary 2/ARIMA-ACF/G20/male-in.tiff]

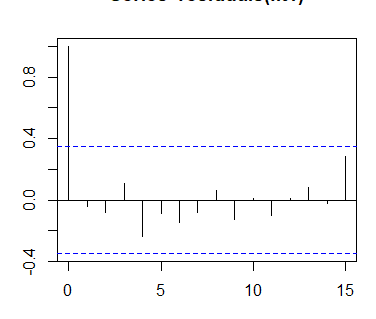

Supplement: Supplementary file 1 [file Data_Sheet_1.zip › Supplementary/Supplementary 2/ARIMA-ACF/G20/male-pr.tiff]

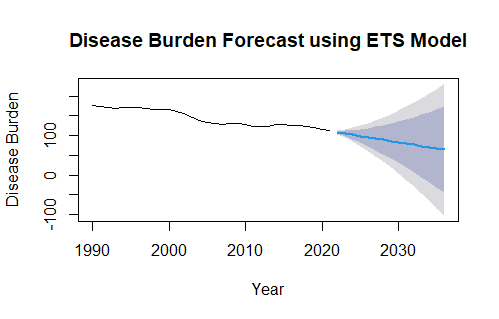

Supplement: Supplementary file 1 [file Data_Sheet_1.zip › Supplementary/Supplementary 2/ETS/China/female-DALY.tiff]

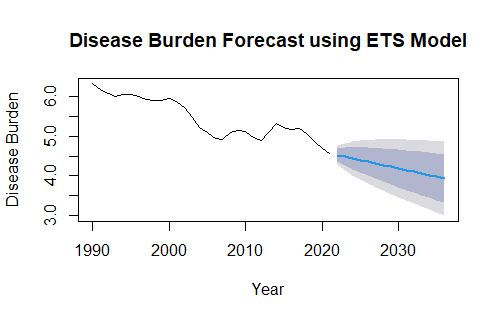

Supplement: Supplementary file 1 [file Data_Sheet_1.zip › Supplementary/Supplementary 2/ETS/China/female-de.tiff]

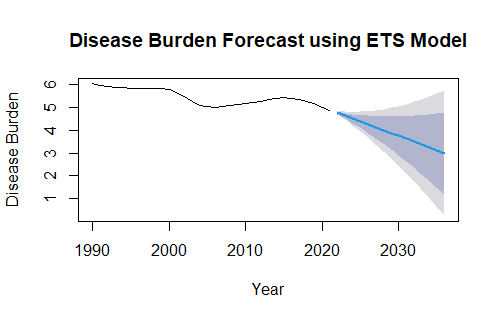

Supplement: Supplementary file 1 [file Data_Sheet_1.zip › Supplementary/Supplementary 2/ETS/China/female-in.tiff]

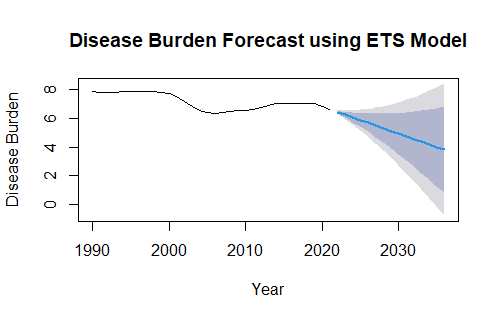

Supplement: Supplementary file 1 [file Data_Sheet_1.zip › Supplementary/Supplementary 2/ETS/China/female-pr.tiff]

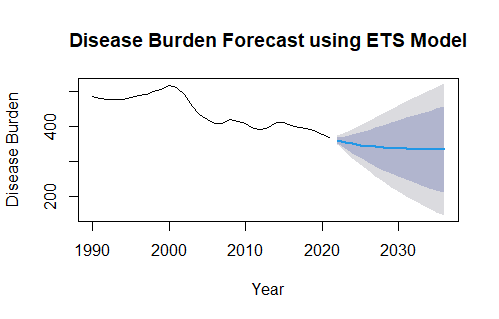

Supplement: Supplementary file 1 [file Data_Sheet_1.zip › Supplementary/Supplementary 2/ETS/China/male-DALY.tiff]

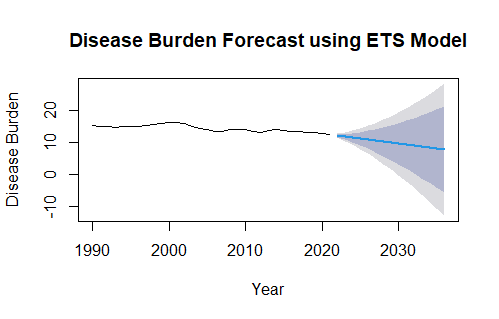

Supplement: Supplementary file 1 [file Data_Sheet_1.zip › Supplementary/Supplementary 2/ETS/China/male-de.tiff]

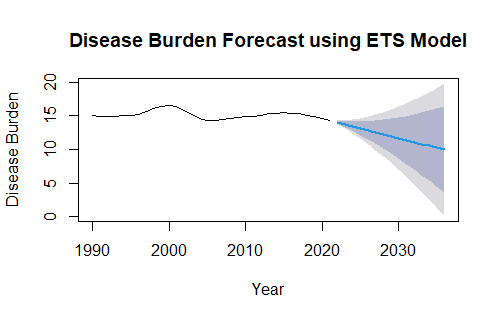

Supplement: Supplementary file 1 [file Data_Sheet_1.zip › Supplementary/Supplementary 2/ETS/China/male-in.tiff]

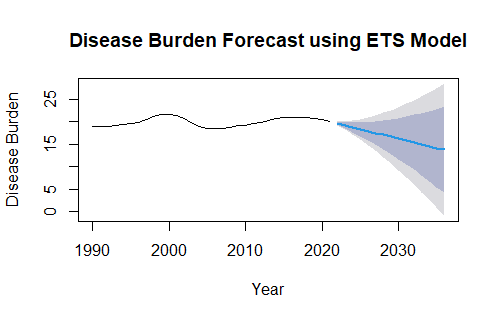

Supplement: Supplementary file 1 [file Data_Sheet_1.zip › Supplementary/Supplementary 2/ETS/China/male-pr.tiff]

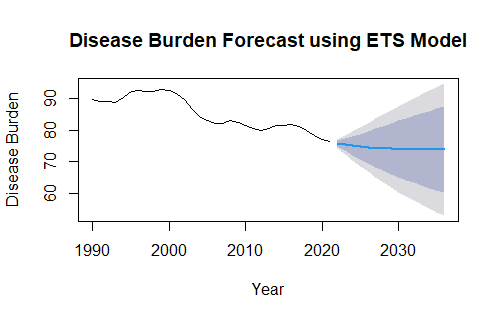

Supplement: Supplementary file 1 [file Data_Sheet_1.zip › Supplementary/Supplementary 2/ETS/G20/female-DALY.tiff]

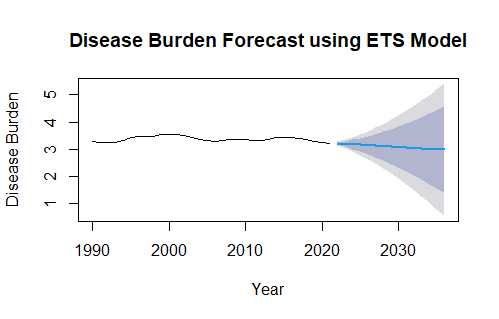

Supplement: Supplementary file 1 [file Data_Sheet_1.zip › Supplementary/Supplementary 2/ETS/G20/female-de.tiff]

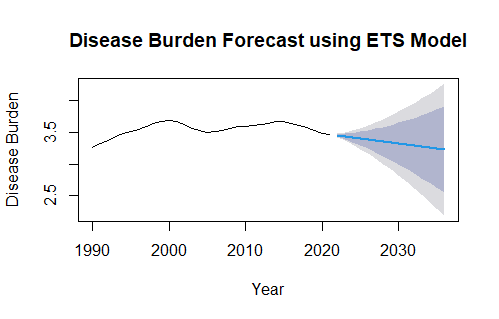

Supplement: Supplementary file 1 [file Data_Sheet_1.zip › Supplementary/Supplementary 2/ETS/G20/female-in.tiff]

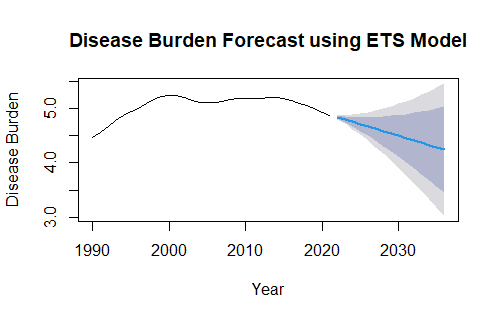

Supplement: Supplementary file 1 [file Data_Sheet_1.zip › Supplementary/Supplementary 2/ETS/G20/female-pr.tiff]

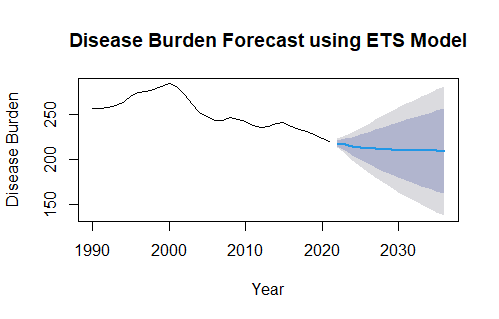

Supplement: Supplementary file 1 [file Data_Sheet_1.zip › Supplementary/Supplementary 2/ETS/G20/male-DALY.tiff]

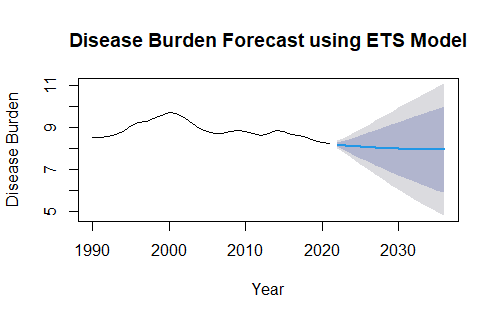

Supplement: Supplementary file 1 [file Data_Sheet_1.zip › Supplementary/Supplementary 2/ETS/G20/male-de.tiff]

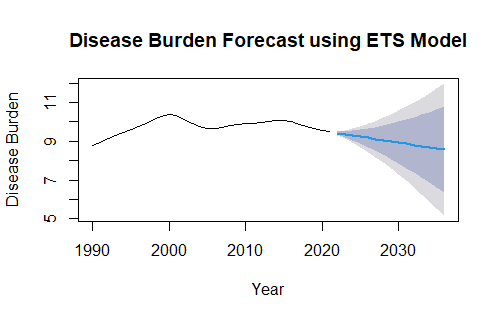

Supplement: Supplementary file 1 [file Data_Sheet_1.zip › Supplementary/Supplementary 2/ETS/G20/male-in.tiff]

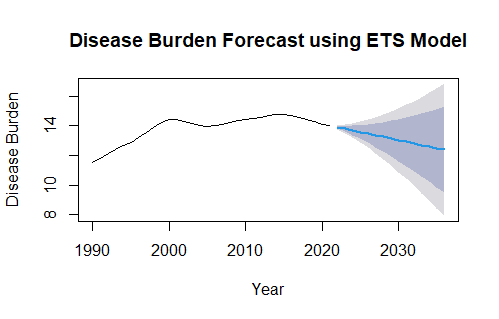

Supplement: Supplementary file 1 [file Data_Sheet_1.zip › Supplementary/Supplementary 2/ETS/G20/male-pr.tiff]

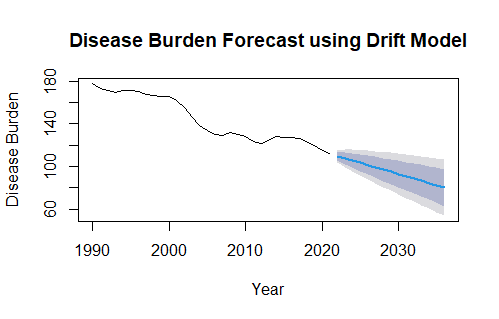

Supplement: Supplementary file 1 [file Data_Sheet_1.zip › Supplementary/Supplementary 2/naive drift/China/female-DALY.tiff]

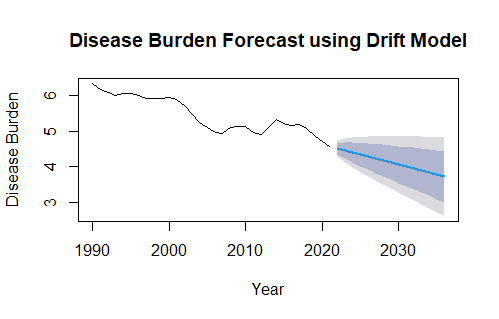

Supplement: Supplementary file 1 [file Data_Sheet_1.zip › Supplementary/Supplementary 2/naive drift/China/female-de.tiff]

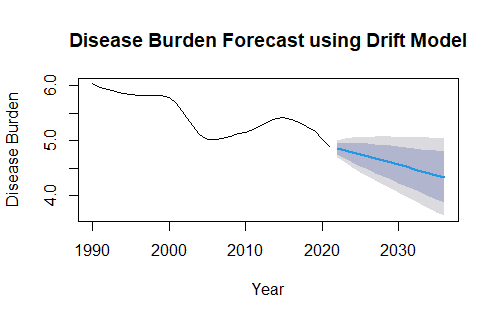

Supplement: Supplementary file 1 [file Data_Sheet_1.zip › Supplementary/Supplementary 2/naive drift/China/female-in.tiff]

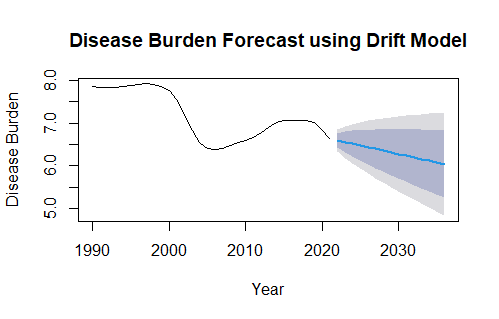

Supplement: Supplementary file 1 [file Data_Sheet_1.zip › Supplementary/Supplementary 2/naive drift/China/female-pr.tiff]

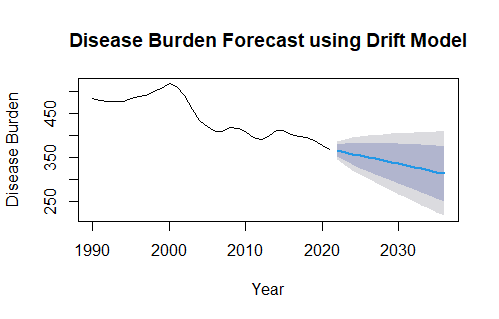

Supplement: Supplementary file 1 [file Data_Sheet_1.zip › Supplementary/Supplementary 2/naive drift/China/male-DALY.tiff]

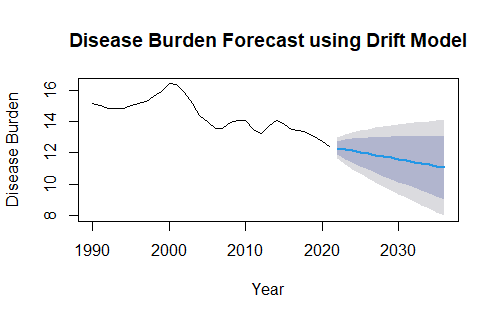

Supplement: Supplementary file 1 [file Data_Sheet_1.zip › Supplementary/Supplementary 2/naive drift/China/male-de.tiff]

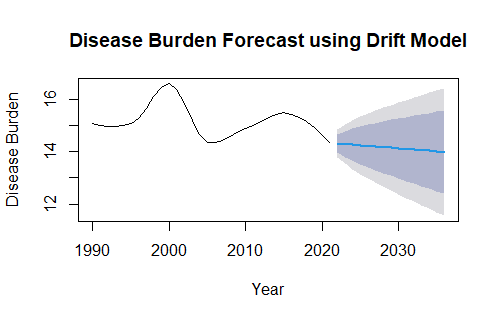

Supplement: Supplementary file 1 [file Data_Sheet_1.zip › Supplementary/Supplementary 2/naive drift/China/male-in.tiff]

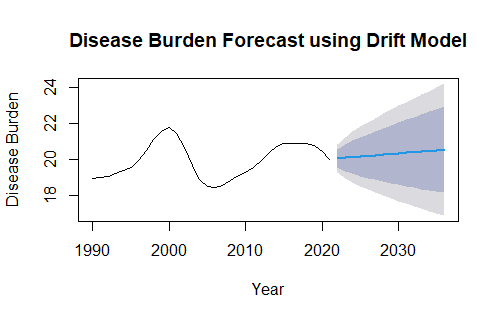

Supplement: Supplementary file 1 [file Data_Sheet_1.zip › Supplementary/Supplementary 2/naive drift/China/male-pr.tiff]

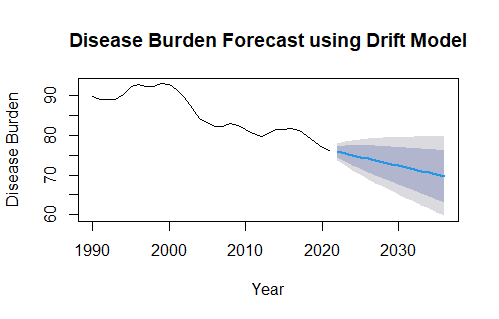

Supplement: Supplementary file 1 [file Data_Sheet_1.zip › Supplementary/Supplementary 2/naive drift/G20/female-DALY.tiff]

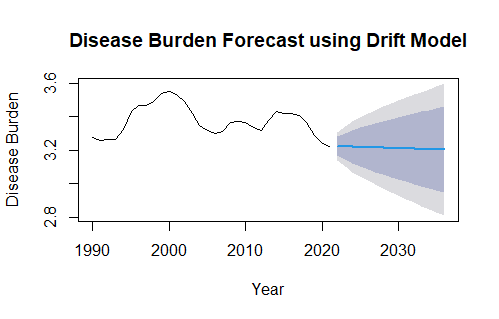

Supplement: Supplementary file 1 [file Data_Sheet_1.zip › Supplementary/Supplementary 2/naive drift/G20/female-de.tiff]

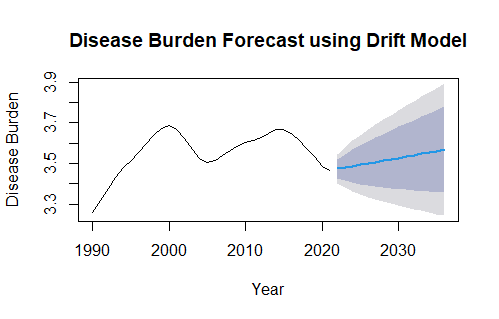

Supplement: Supplementary file 1 [file Data_Sheet_1.zip › Supplementary/Supplementary 2/naive drift/G20/female-in.tiff]

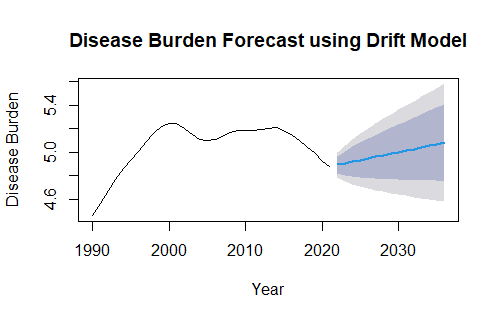

Supplement: Supplementary file 1 [file Data_Sheet_1.zip › Supplementary/Supplementary 2/naive drift/G20/female-pr.tiff]

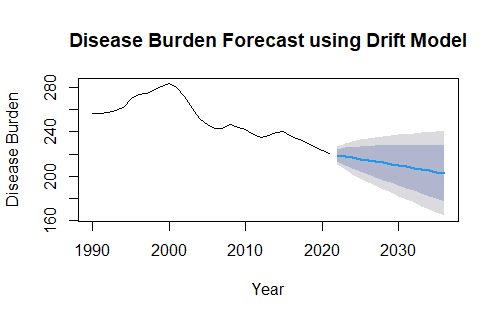

Supplement: Supplementary file 1 [file Data_Sheet_1.zip › Supplementary/Supplementary 2/naive drift/G20/male-DALY.tiff]

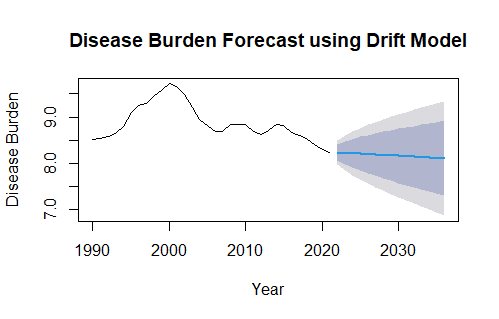

Supplement: Supplementary file 1 [file Data_Sheet_1.zip › Supplementary/Supplementary 2/naive drift/G20/male-de.tiff]

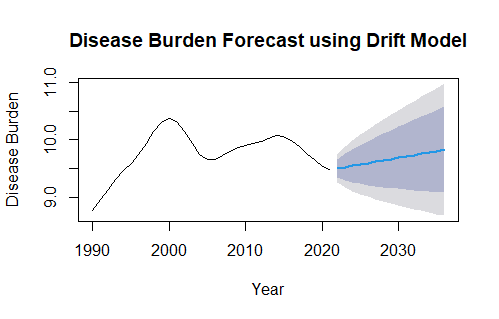

Supplement: Supplementary file 1 [file Data_Sheet_1.zip › Supplementary/Supplementary 2/naive drift/G20/male-in.tiff]

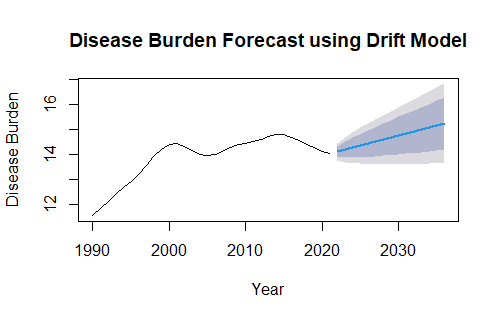

Supplement: Supplementary file 1 [file Data_Sheet_1.zip › Supplementary/Supplementary 2/naive drift/G20/male-pr.tiff]

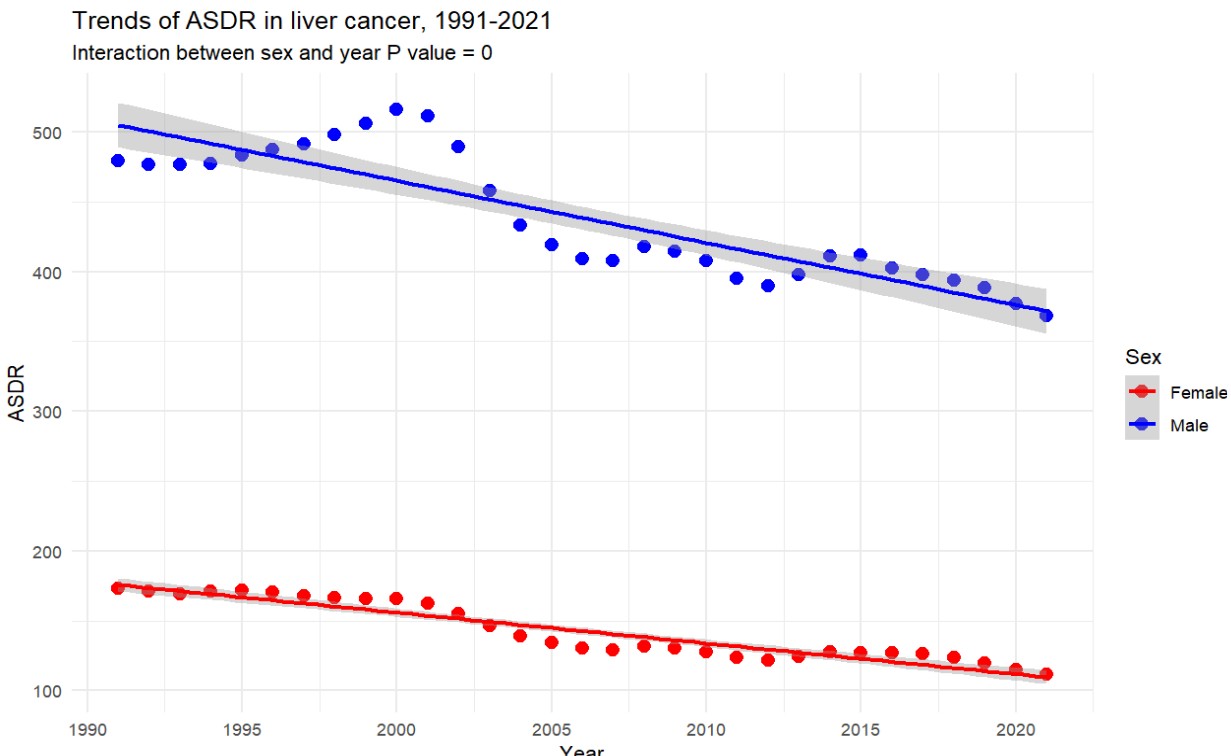

Supplement: Supplementary file 1 [file Data_Sheet_1.zip › Supplementary/Supplementary 4/China-ASDR.jpg]

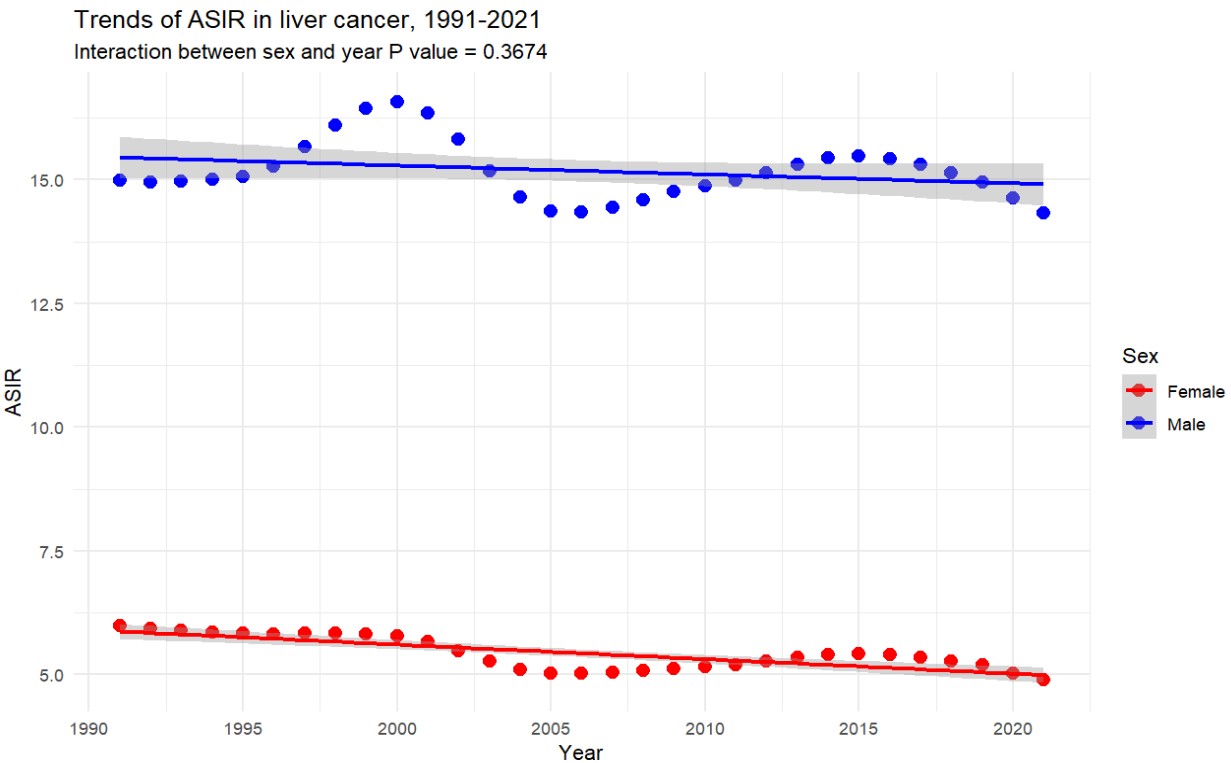

Supplement: Supplementary file 1 [file Data_Sheet_1.zip › Supplementary/Supplementary 4/China-ASIR.jpg]

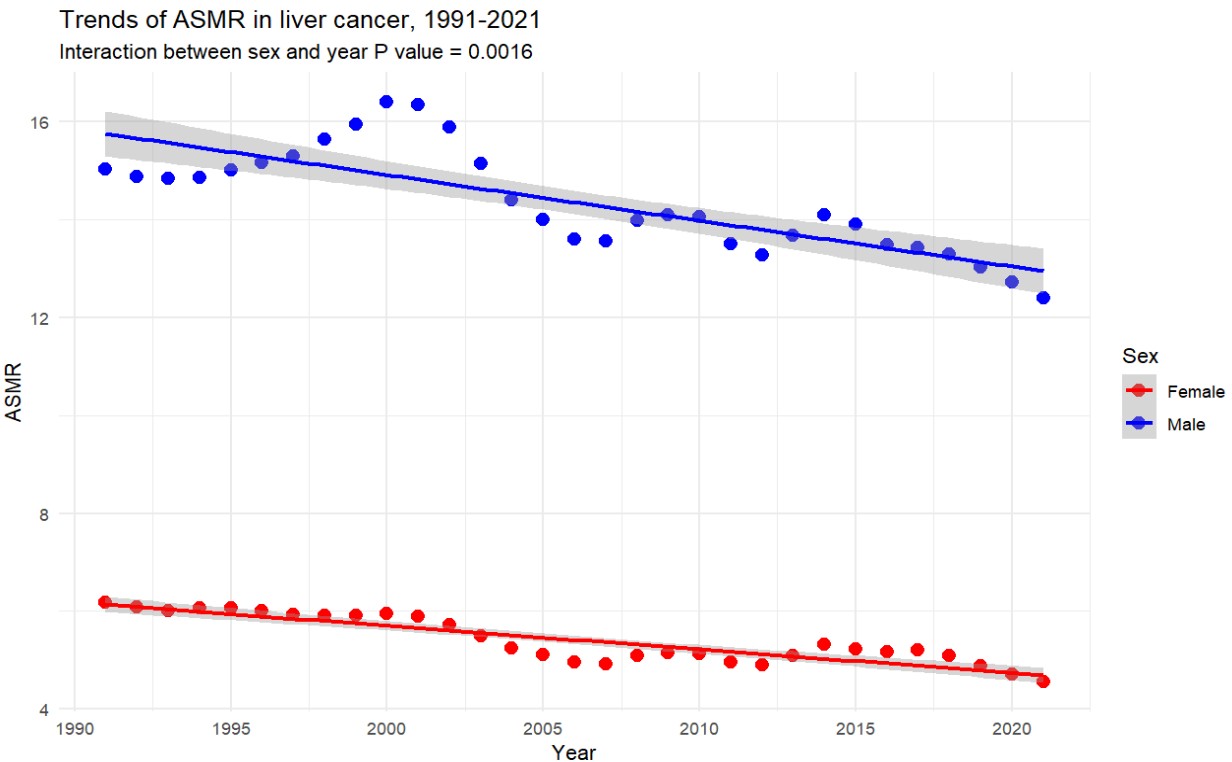

Supplement: Supplementary file 1 [file Data_Sheet_1.zip › Supplementary/Supplementary 4/China-ASMR.jpg]

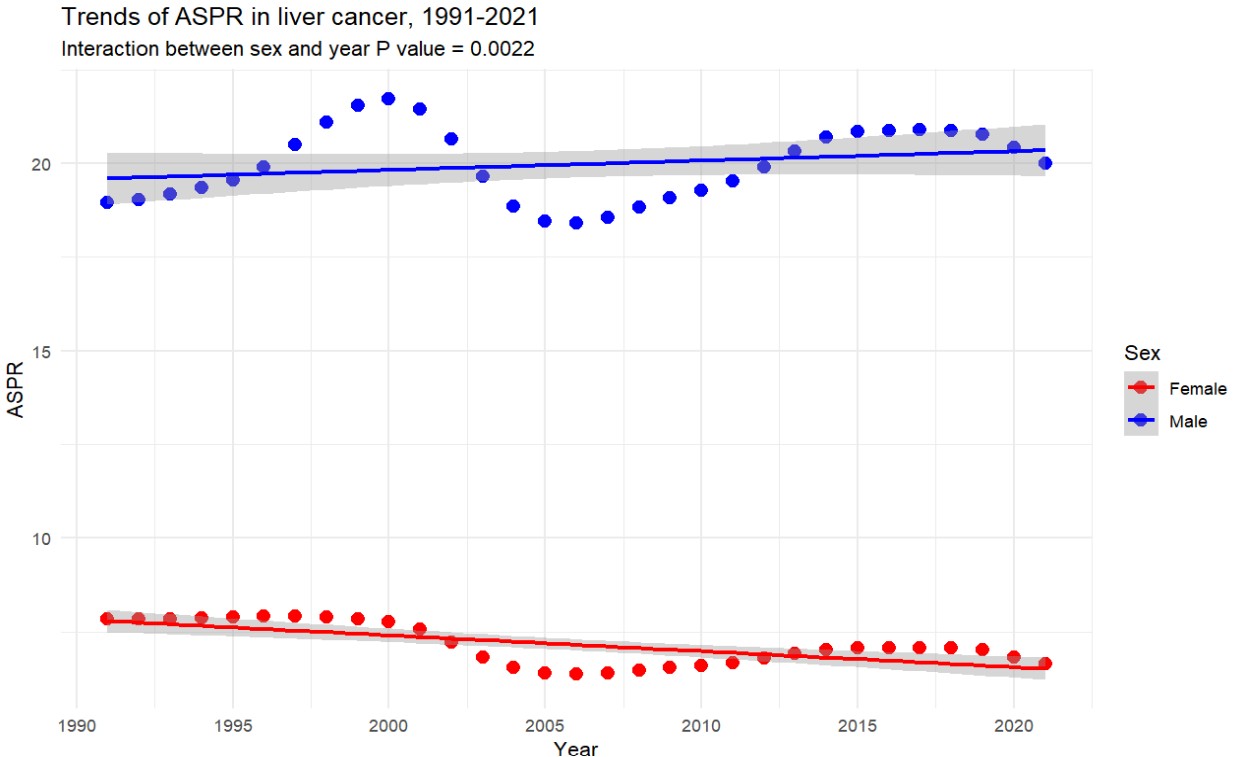

Supplement: Supplementary file 1 [file Data_Sheet_1.zip › Supplementary/Supplementary 4/China-ASPR.jpg]

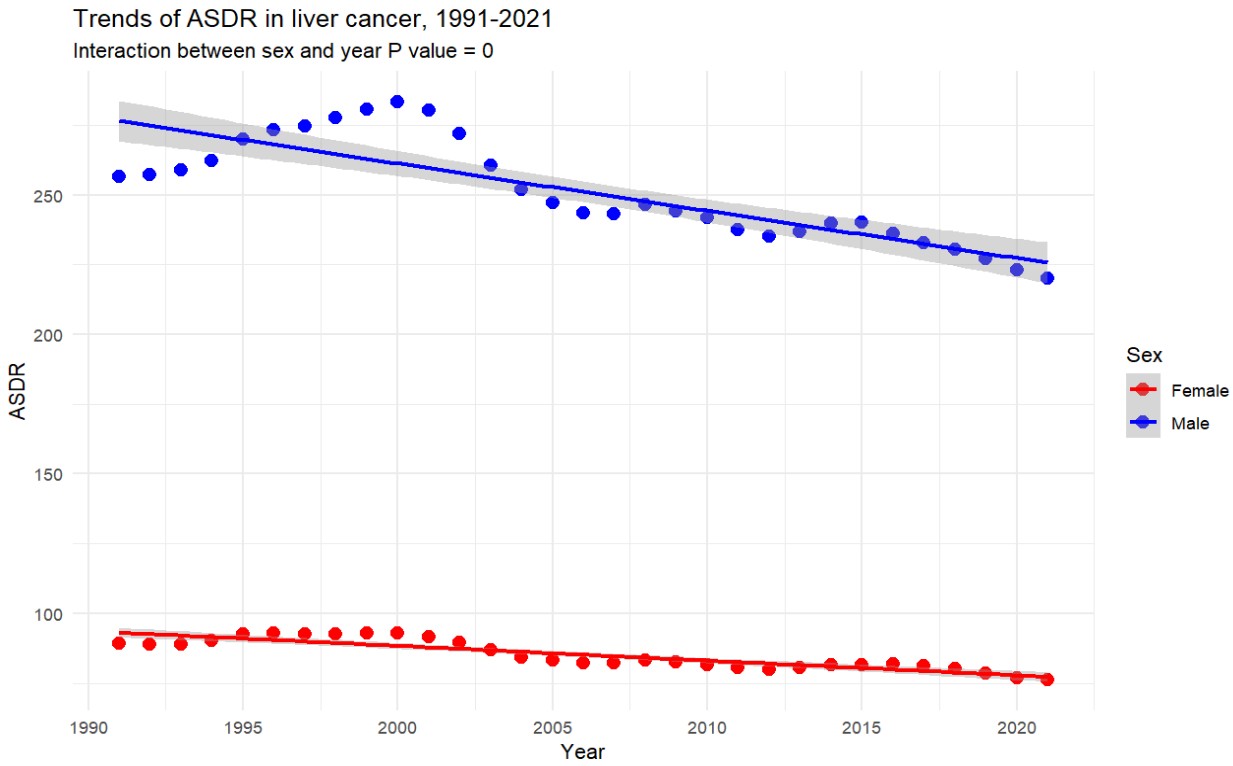

Supplement: Supplementary file 1 [file Data_Sheet_1.zip › Supplementary/Supplementary 4/G20-ASDR.jpg]

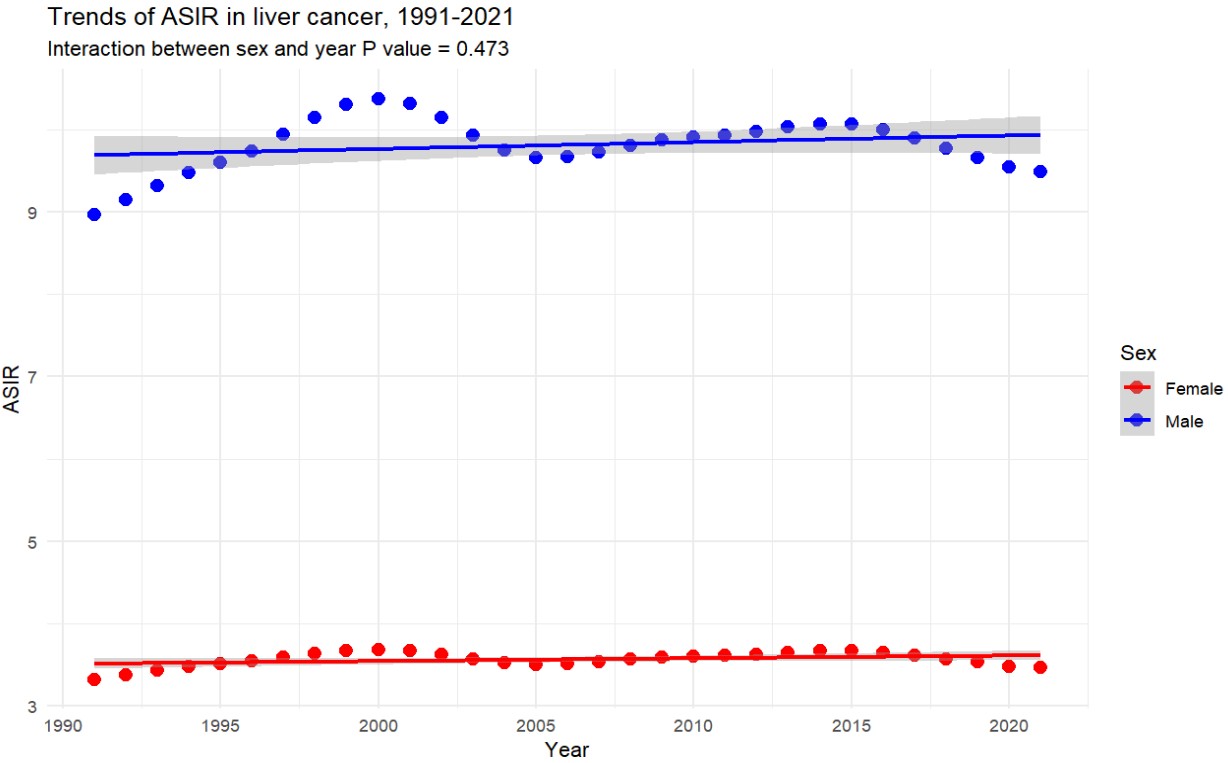

Supplement: Supplementary file 1 [file Data_Sheet_1.zip › Supplementary/Supplementary 4/G20-ASIR.jpg]

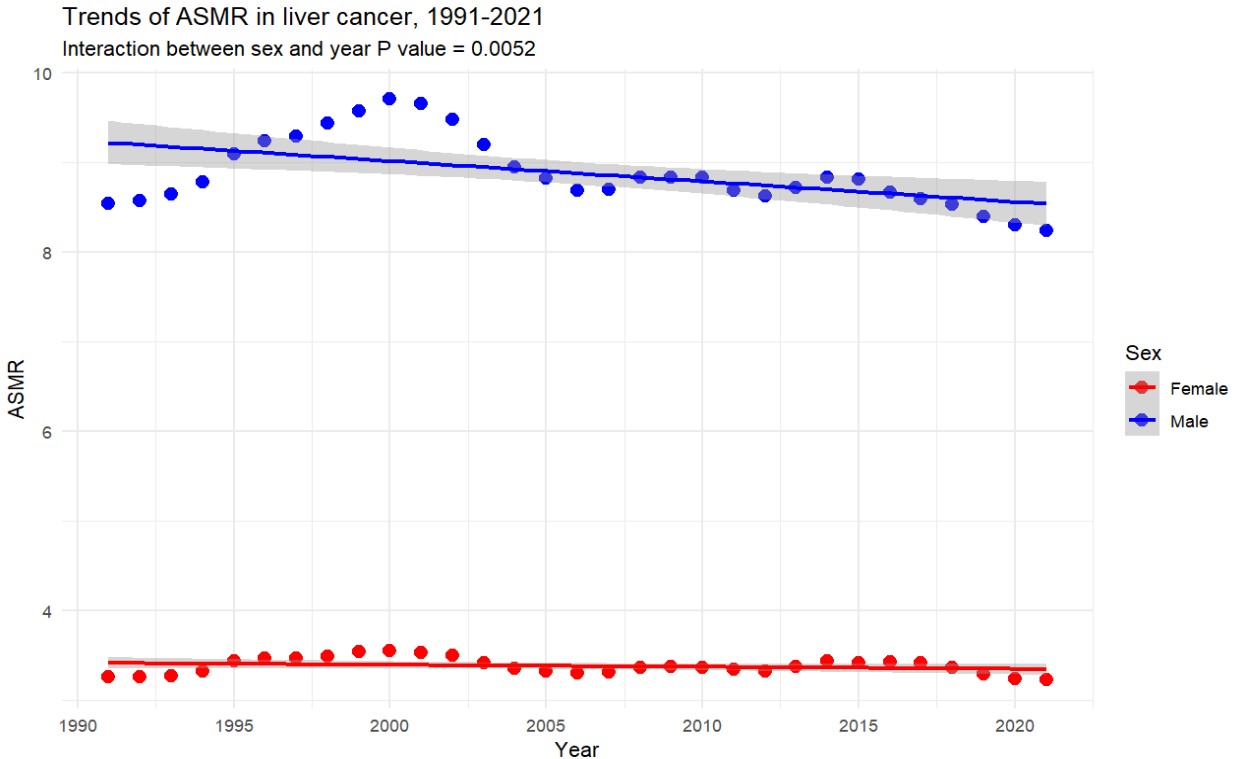

Supplement: Supplementary file 1 [file Data_Sheet_1.zip › Supplementary/Supplementary 4/G20-ASMR.jpg]

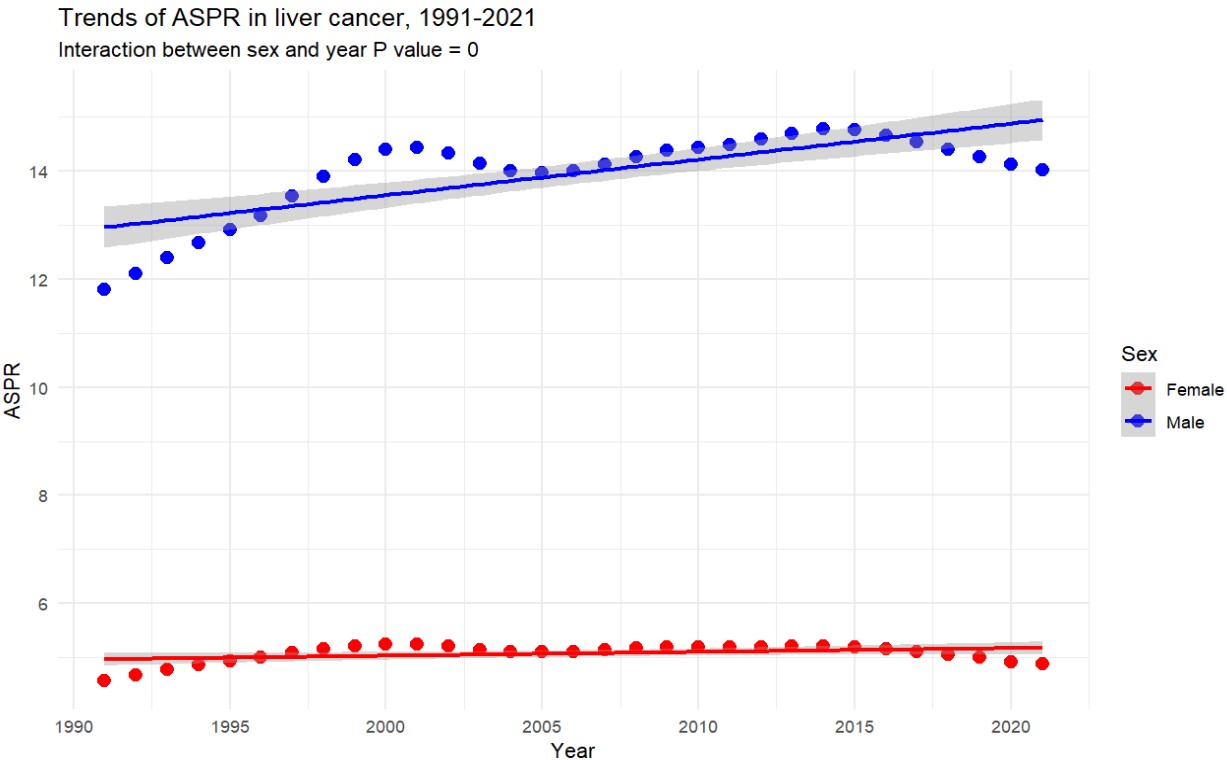

Supplement: Supplementary file 1 [file Data_Sheet_1.zip › Supplementary/Supplementary 4/G20-ASPR.jpg]
